# IFNγ preconditioning improves neuroprotection of MSC-derived vesicles on injured retinal ganglion cells by suppressing microglia activation via miRNA-dependent ribosome activity

**DOI:** 10.20517/evcna.2024.66

**Published:** 2025-02-19

**Authors:** Tianjing You, Yuanxing Yang, Luodan A, Xuan Cheng, Xi Lin, Qingle Liang, Lingling Ge, Jing Xie, Siyu Chen, Na Liu, Juncai He, Haiwei Xu, Xiang Ma

**Affiliations:** ^1^Department of Ophthalmology, The First Affiliated Hospital of Dalian Medical University, Dalian 116014, Liaoning, China.; ^2^Southwest Eye Hospital, Southwest Hospital, Third Military Medical University (Amy Medical University), Chongqing 400038, China.; ^3^Key Lab of Visual Damage and Regeneration and Restoration of Chongqing, Southwest Eye Hospital, Southwest Hospital, Chongqing 400038, China.; ^4^Department of Ophthalmology, The 920 Hospital of PLA Joint Logistics Support Force, Kunming 650032, Yunnan, China.; ^#^Authors contributed equally.

**Keywords:** Optic nerve crush, small extracellular vesicles, interferon gamma, interferon-responsive microglia, microRNAs, ribosome activity

## Abstract

**Aim:** Microglial activation plays a pivotal role in the pathogenesis of retinal ganglion cell (RGC) degeneration resulting from optic nerve crush (ONC). Small extracellular vesicles (sEVs) secreted by mesenchymal stem cells (MSCs) have the potential to prevent retinal degeneration by modulating microglial activation. In this study, we elucidated the specific effects of sEVs derived from IFN-γ-primed MSCs on the phenotypic transition of microglia and the associated pathways in ONC mice.

**Methods:** The ONC mice model was established and administered intravitreal injection with the sEVs derived from native MSCs (native sEVs) and the sEVs derived from MSCs primed with IFN-γ (IFNγ-sEVs). Their respective effects on the survival of the retinal ganglion cells (RGCs) and the transition of microglia phenotypes were determined through visual function testing and immunohistochemical staining. Combined with mRNA seq and microRNA seq techniques, we elucidated the mechanism of modulation of microglia phenotypic transformation by sEVs derived from MSCs primed by IFNγ.

**Results:** It demonstrated that IFNγ-sEVs exhibited superior protective effects against RGC loss and reduced inflammatory responses in the ONC retina compared to native sEVs. Both types of sEVs promoted microglia activation to disease-associated microglia (DAM) phenotype, while IFNγ-sEVs especially suppressed interferon-responsive microglia (IRM) activation during RGCs degeneration. Subsequent miRNA sequencing suggested that *miR-423-5p*, which exhibited the most significant differential expression between the two sEVs types and elevated expression in IFNγ-sEVs, inhibited the expression of IRM and ribosomal genes.

**Conclusion:** These findings suggest that IFN-γ-preconditioned MSCs may enhance sEVs of neuroprotection on RGCs by suppressing IRM activation through the secretion of sEVs containing specific microRNAs in ONC mice.

## INTRODUCTION

Visual information is transmitted from the retina to the optic center of the cerebral cortex in the brain via the optic nerve, which is formed by a bundle of axons emerging from the retinal ganglion cells (RGCs)^[1]^. The injury and loss of RGCs constitute a primary cause of visual impairment and blindness. RGC soma and axon damage have been observed in the early stage of glaucoma, optic nerve transection (ONT), compression, and intracranial hypertension, which results in optic nerve atrophy, hypofunction, RGC loss, and even blindness^[2]^. However, there are currently few effective neuroprotective approaches to maintaining RGCs in the clinic. To address this challenge, we used the optic nerve crush (ONC) model and found that 80% of RGCs in mice are lost 2 weeks after injury^[3]^.

Mesenchymal stem cells (MSCs) are defined by their neuroprotective, immunomodulatory, and antioxidant properties, promising for treating RGC degeneration^[4]^. Transplanted MSCs usually migrate to injured tissues and promote a protective microenvironment; the therapeutic effects of MSCs are largely attributed to paracrine effectors including secreting cytokines, inflammatory mediators, extracellular matrix components, and proteins^[5]^. Among such effectors, small extracellular vesicles (sEVs), with a diameter ranging from ~ 30 to less than 200 nm, exhibit beneficial effects on injured RGCs comparable to transplanted MSCs, whereas producing lower immunogenicity and risk for tumorigenicity^[6]^. The biomolecules loaded by sEVs, including proteins, metabolites, and nucleic acids (noncoding RNAs, mRNA, and DNA), depend on the cellular and environmental context of the parent cell^[7]^. Thus, preconditioning the MSCs with the specific stimulus usually enhances the therapeutic activities of sEVs^[7]^. Preconditioning MSCs with interferon-gamma (IFN-γ), a critical cytokine regulating innate immunity against viruses and bacteria, stimulates sEVs enriched in material involved in regulating interferon responses and inflammatory pathways^[8,9]^. These signaling pathways directly participate in the degeneration and regeneration of RGCs^[10-12]^. Therefore, we further investigated whether IFN-γ preconditioning effectively enhanced the neuroprotective effects of MSC-derived sEVs on RGCs in the ONC mice and the underlying mechanisms.

Microglia are highly specialized predominant immune cells and reside in the outer plexiform layers (OPL), the inner plexiform layers (IPL), and the ganglion cell layer (GCL) of the retina^[13,14]^. Following ONC, the microglia are activated and adopt a reactive phenotype that is characterized by enlarged cell bodies and shortened processes, and play a central player in the degeneration of RGCs^[12]^. Modulation of microglial activation and switching them from a detrimental to a beneficial phenotype using the MSC-derived sEVs represent a potential therapeutic method for treating central nervous system (CNS) injury^[15,16]^. However, almost all the above studies frame microglia phenotypes into traditionally dichotomic categories using outdated terms such as “resting *vs*. activated”or “M1 *vs*. M2” the label being recommended strictly avoiding^[15,17]^. Recently, an increasing number of studies based on novel single-cell/nuclear sequencing and multi-omics analysis, have shown that microglia respond to multiple insults and are activated into various novel disease-stage-specific microglia, thereby exerting different roles in the immunopathogenesis of neurodegenerative diseases^[18]^. A recent study using single-cell RNA sequence has identified new microglia subpopulations involved in the development of ONC, based on their distinct gene expression profiles. These include disease-associated microglia (DAM) and type I interferon (IFN-I)-responsive microglia (IRM)^[19]^, which have been suggested to exacerbate or ameliorate the pathological changes of neurodegenerative diseases^[20,21]^. However, to date, the effect of MSC-derived sEVs on the activation of DAM and IRM and the corresponding pathways remains to be determined.

In the present study, we explored the modulation of MSC-derived sEVs on the microglial phenotypic activation and its contribution to protecting injured RGCs in ONC. For this purpose, we administered the sEVs derived from native MSCs (native sEVs) and the sEVs derived from MSCs primed with IFN-γ (IFNγ-sEVs) through intravitreal injection immediately after ONC. Then, we compared their effects on RGC survival and the activation of DAM or IRM. To validate the underlying mechanisms of sEVs regulating microglial phenotypes, RNA sequencing in LPS + IFNγ-activated BV2 cell line treated with the vehicle, native sEVs, or IFNγ-sEVs, was used to detect differentially expressed genes (DEGs) and screen out the most significant pathways. Ultimately, miRNA profiling of sEVs suggested that a set of differentially expressed miRNAs between native sEVs and IFNγ-sEVs might play a key role in inhibiting the IRM activation by decreasing ribosome activity. Collectively, our findings revealed a novel role for MSC-derived sEVs in microglial phenotypes and their underlying molecular mechanisms, highlighting the potential of sEVs as a promising neuroprotective treatment to halt the progression of neurodegenerative diseases by redirecting microglia to beneficial and neuroprotective phenotypes.

## METHODS

### Animals

ENSIWEIER Biotech Ltd, located in Chongqing, China, provided C57BL/6 mice aged 6 to 8 weeks. At the Southwest Hospital Animal Center, these animals were housed in a strictly controlled, pathogen-free setting. The mice were provided with a standard nutritional regimen and had unrestricted access to water, while being exposed to a 12-h light and dark cycle. For euthanasia, a 30% chamber replacement rate of CO_2_ was utilized. The animal study protocol was granted approval by the Animal Welfare and Ethics Committee at the Third Military Medical University (Project title: Effect and mechanism of endogenous stem cell activation *in situ* and microenvironmental regulation on retinal regeneration and repair of optic nerve; Approval date: 2021.08.23; Approval number: AMUWEC20211860). The study was conducted in accordance with the ethical standards and welfare requirements for animals.

### ONC model

Tribromoethanol anesthesia was induced (0.2 mL/10g each) in C57 mice and followed with oxybuprocaine hydrochloride eye drops. After a brief 10-s pause, the optic nerve was exposed by cutting from the outer canthus of the orbit to the deepest layer of the conjunctival tissue. The venous sinus should be avoided when separating the tissue. Then, the optic nerve was injured by clamping it with reverse surgical forceps for 10 s at 2 mm from the end of the optic nerve. The experiment was limited to mice with no obvious postoperative fundus hemorrhage and ischemia.

### Flash visual evoked potentials recording

The detection for visual electrophysiological diagnosis complied with the guidelines set by the International Society for Clinical Electrophysiology of Vision (ISCEV). After a 15-min dark adaptation period, mice received intraperitoneal anesthesia with tribromoethanol, dosed at 0.2ml/10g of body weight. Flash visual evoked potentials (F-VEP) was performed by inserting silver needle electrodes beneath the skin at the midpoint of both ears. The reference electrode was situated on the same side cheek, and the ground electrode was attached to the tail of the mice. White flash stimuli were delivered at a rate of 1.5 Hz and superimposed 50 times. Each eye was recorded three times for stable waveforms, with the contralateral eye covered by a shadow. The assessed metrics encompassed the F-VEP latency (the time of reaching the P1 wave peak, measured in milliseconds) and the N1-P1 amplitude (the voltage difference from the N1 wave to the P1 wave peak, recorded in millivolts). All data points were systematically collected by a computer, and the average of the three measurements was computed.

### Immunohistochemistry

Frozen tissue slices were immunofluorescence stained in accordance with the previously mentioned protocol^[13]^. After a 15-min fixation in a 4% paraformaldehyde solution at 4 °C, the eyecups were then immersed in a 30% sucrose solution and stored overnight at 4 °C. The retinas were subsequently encased in an optimal cutting temperature (OCT) medium (Sakura FineTek, Torrance, CA, USA) and sliced sagittally into 14 µm sections using a cryotome. The sections were permeabilized and blocked using a solution that consists of phosphate-buffered saline (PBS) with 0.1% Triton X-100 and 10% donkey or goat serum. The sections were treated with primary antibodies [Supplementary Table 1] overnight at 4 °C in a solution of PBS containing 0.03% Triton X-100 and 5% donkey or goat serum. After five washes with PBS, the sections were subsequently incubated with fluorophore-conjugated secondary antibodies for 60 min at 37 °C. In the end, the nuclei were labeled with DAPI (Sigma-Aldrich).

In accordance with the previous protocol^[22]^, retinal flat mounts were performed with fluorescence staining. To get each retina completely permeabilized, intact retinas from the ONC mice were treated with PBS containing 0.5% Triton X-100 at 70 °C for 15 min, followed by a 15-min rinse at room temperature in the same solution. Then, the retinas were stored overnight at 4 °C with primary antibodies diluted in the specialized buffer of 2% bovine serum albumin and 2% Triton X-100. The next day, the retinas underwent three 10-min washes in PBS, followed by a 2-h incubation with secondary antibodies in the same buffer at room temperature. Finally, immunofluorescence staining images were obtained using a Zeiss LSM 780 confocal microscope.

### Analysis of immunofluorescence staining image

As previously described^[14,22]^, sections of the retina that intersect with the optic nerve were selected. Regions on either side of the optic nerve head were selected at equal intervals from the center outwards, and the number of positive cells in these regions was tallied. The retinal flat mount was divided into four quadrants, and immunofluorescence staining images of Rbpms were taken at 400x magnification in three different regions of each quadrant. The cell counts were averaged across five eyes per group. The quantification and structural examination of retinal microglia were conducted following a previously established methodology^[13]^. The morphological assessment involved counting the grid intersection points and the number of processes for each microglial cell. Confocal imaging was performed at a resolution of 135 µm × 135 µm, with z-steps of 0.2 µm, to evaluate microglial phagocytic ability. The major and minor axes of each cell were manually delineated and measured according to the protocol described by McWhorter *et al.*^[23]^. The primary dimension was identified as the cell’s greatest length, whereas the secondary dimension was the perpendicular measurement through the nucleus. The elongation factor was calculated by the ratio of the long axis to the short axis.

### Hematoxylin and eosin staining

The whole eyeball was preserved in a 4% PFA solution and made slices with a thickness of 5 µm. According to the protocol provided by Beyotime (C0105M), slices were stained with hematoxylin and eosin (H&E). Images of the whole sections were obtained at high resolution and the cells in the GCL of each 250 μm region were analyzed and counted with the assistance of Case Viewer 2.6 software.

### Cell culture

As in our previous study^[24]^, 21-day-old RCS-rdy+ rats had their femurs and tibias carefully isolated and washed with PBS. The bone marrow was then extracted from the cavities and the cells were cultivated in a complete medium (RAXMX-90011, Orilcell) designed for bone marrow mesenchymal cells. These cells were maintained in a humidified incubator set at 37 °C with 5% CO_2_. After two days, the culture medium was refreshed to remove any non-adherent cells. The MSCs reached approximately 80%-90% confluency and were then passaged to the next generation. Cells from the third passage were used in subsequent experiments.

BV2 microglial cells were maintained at 37 °C in a 5% CO_2_ incubator, using Dulbecco’s Modified Eagle Medium (SH30023.01B, Hyclone) enriched with 10% fetal bovine serum (FB25015, Clark Bioscience).

### sEVs isolation

Previously described methods were used for the isolation of sEVs^[24]^. The medium was supplemented with 50 ng/mL recombinant rat IFNγ (598806 Biolegend) and incubated for 48 h to pretreat MSCs. To obtain sEVs, the serum in the medium was replaced with sEV-depleted FBS (C38010050, VivaCell). Culture supernatants from MSCs grown in sEV-free media, either pretreated with IFNγ or not, were collected after 48 h of culture. Supernatants were centrifuged over 1,250 rpm for 10 min at 4 ºC and over 3,500 rpm for 15 min at 4 ºC to remove cell debris, and placed in the centrifuge at 100,000 × g for 70 min at 4 ºC with Beckman OPTI-MA XPN-100 and SOV28Ti Swinging Bucket Rotor to isolate the EVs. sEVs were achieved by passing the EVs through a 0.22 μm filter to eliminate microvesicles. For further purifications, the sEVs were then centrifuged for 70 min at 100,000 × g and sediment collected. Finally, the pellet was resuspended in PBS, and the sEVs were labeled with PKH26 (Sigma-Aldrich, MINI26) according to the manufacturer’s instructions.

### Transmission electron microscopy

After dilution in PBS, sEVs were isolated from the bone marrow stromal cells (BMSCs) culture supernatant and placed onto formvar-coated copper grids, where they were allowed to settle at room temperature for 20 min. The samples were then fixed by immersing them in 0.05 M phosphate buffer containing 2% polyformaldehyde solution and 2% glutaraldehyde for 2 min. Afterward, the samples were cleaned 3 times with distilled water. The copper grids were then stained with 1% phosphotungstic acid for 1 min. Once dried, images of the samples were taken using a transmission electron microscope (JEM-1400PLUS, Japan).

### Nanoparticle tracking analysis

sEVs were extracted from the supernatant of equally and simultaneously cultured cells in the native MSCs group and the IFNγ-primed MSCs group. Three supernatant samples per group were used, and experiments were repeated at least three times. Diluted sEVs with PBS were drawn into a 1 mL syringe. Following the manufacturer’ guidelines for the ZetaView instrument (Particle Metrix, Germany), the sEVs were injected into the analysis chamber. The dynamic image was then captured and examined.

### Intravitreal injection

The mice were arbitrarily divided into three categories: the ONC group, the native-sEVs-treated group, and the IFNγ-sEVs-treated group. Initially, the two types of sEVs were diluted to a standardized concentration of 10^9^ particles. Following ONC, the treated group was administered intravitreal injections of 1 µL of either native sEVs or IFNγ-sEVs. The ONC group received an equal volume of PBS into the vitreous as a control.

### mRNA and microRNA sequencing

For mRNA sequencing, retinal samples were collected from three groups: ONC, Native-sEVs-treated, and IFNγ-sEVs-treated. Extracted total RNA utilizing a Trizol reagent kit (Invitrogen, Carlsbad, CA, USA) according to the manufacturer’s guidelines. Assessed the RNA quality via an Agilent 2100 Bioanalyzer (Agilent Technologies, Palo Alto, CA, USA) and confirmed its integrity through RNase-free agarose gel electrophoresis. Employed Oligo (dT) beads to isolate eukaryotic mRNA, followed by breaking down the mRNA into smaller fragments using a fragmentation buffer. Transformed the fragmented mRNA into cDNA utilizing the NEBNext Ultra RNA Library Prep Kit for Illumina (NEB #7530, New England Biolabs, Ipswich, MA, USA). Conducted end-repair on the purified double-stranded cDNA, appended an adenine residue, and ligated them to Illumina sequencing adapters. Purified the ligation products with AMPure XP Beads (1.0X) and then amplified the fragments through PCR. Ultimately, sequenced the cDNA library on an Illumina Novaseq6000 platform at Gene Denovo Biotechnology Co. (Guangzhou, China).

After 48 h of culture in sEVs-depleted fetal bovine serum, total RNA was collected from sEVs derived from both native BMSCs and IFN-γ-primed BMSCs for subsequent experiments. MicroRNA sequencing was conducted to assess differences in expression levels between these two sEV sources. The sequencing procedure involved: (1) extraction of total RNA; (2) attachment of 3’ and 5’ adaptors; (3) real-time PCR amplification; (4) purification of the small RNA library using gel electrophoresis; and (5) validation of the library. The microRNA data analysis was conducted with ACGT101-miR (LC Sciences, Houston, Texas, USA) and consisted of: (1) elimination of the 3’ adapter and non-specific sequences; (2) retention of sequences with lengths between 18–26 nucleotides through size selection; (3) utilization of mRNAs for comparative analysis and sequence filtering; (4) refinement to obtain valid data for microRNAs identification; and (5) assessment of differentially expressed microRNAs. A *P*-value of ≤ 0.05 was deemed statistically significant.

### RNA isolation and reverse-transcription quantitative polymerase chain reaction analysis

In accordance with the provided guidelines, total RNA was isolated from BV2 cells utilizing RNAiso Plus (Cat. No. D9108A, Takara), and miRNAs were extracted using the miRNeasy Mini Kit (217004, Qiagen). Subsequently, the RNA samples were converted into cDNA using the PrimeScriptTM RT reagent kit (Cat. No. #RR037A, Takara), and the miRNA samples were processed for cDNA synthesis with the miRNA 1st Strand cDNA Synthesis Kit (MR101-01, Vazyme). GAPDH or U6 levels were used to standardize the results. Supplementary Table 2 contains a list of all primers.

### Statistical analysis

The mean ± standard deviation (SD) was used to display all data. One-way ANOVA followed by Tukey’s multiple comparisons test was used for comparisons of multiple groups and unpaired t-tests were employed to compare two groups. All analyses were performed using GraphPad Prism 8. *P* values < 0.05 were deemed statistically significant.

## RESULTS

### IFNγ preconditioning improved therapeutic effects of MSC-sEVs on RGC degeneration after ONC

**Figure 1:**
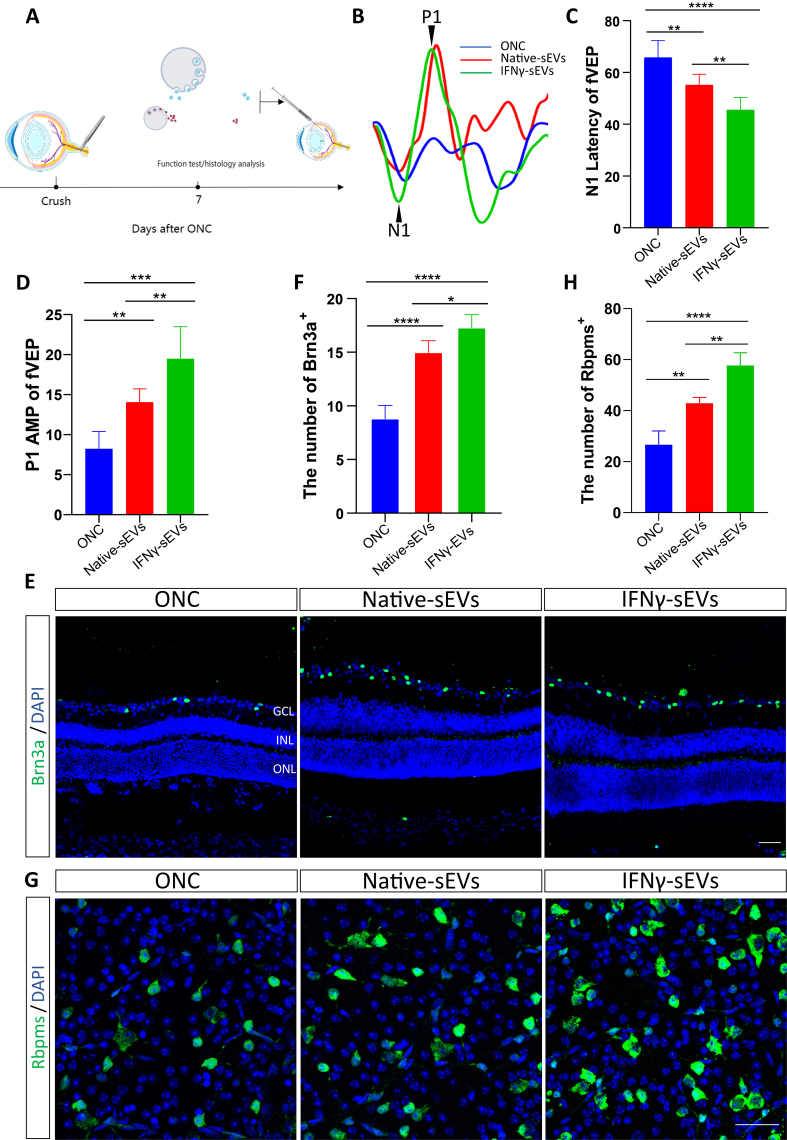
Implantation of sEVs derived from IFNγ-primed MSCs preserves visual function and RGC counts better after ONC. (A) Schematic diagram demonstrating the experimental setup for the model of ONC, followed by sEVs injection in the eye; (B) Representative waveforms of FVEP test 7 days after sEVs implantation; (C and D) P1 amplitudes (C) and N1 latencies (D) at 7 days after ONC and sEVs treatment (*n* = 5 eyes per group); (E) Representative images of retinas from sEVs infusion after ONC, immunohistochemically stained for Brn3a (green) and DAPI (blue). Scale bar, 20 μm; (F) Graph showing the mean number of Brn3a-positive RGCs in retinas (*n* = 5 eyes per group); (G) Representative images of retinal flat mounts stained for Rbpms. Scale bar, 20 μm; (H) Graph showing the mean number of Rbpms-positive RGCs per field in retinal flat mounts (*n* = 5 eyes per group). Using one-way ANOVA (C, D, F and H). Data are shown as mean ± SD. ONC: Optic nerve crush; FVEP: flash visual evoked potential; ONL: outer nuclear layer; IPL: inner plexiform layer; GCL: ganglion cell layer; RGC: retinal ganglion cell; sEVs: small extracellular vesicles; MSCs: mesenchymal stem cells; IFNγ: interferon-gamma; NS: no significant difference. ^*^P < 0.05; ^**^P < 0.01; ^***^P < 0.001; ^****^P < 0.0001.

To gain sEVs, marrow MSCs were isolated from the rat bone, spontaneously showing the potential for osteogenic, adipogenic, and chondrogenic differentiation with a long spindle shape [Supplementary Figure 1A and B]. Then, MSCs were cultured for 48 h under a conditional medium with 50 ng/mL IFN-γ stimulated or without stimulation, and differential centrifugation was used to purify native sEVs and IFNγ-sEVs from MSCs. Both the two sEVs were cup-shaped spherical vesicles in transmission electron microscopy (TEM) images [Supplementary Figure 1C]. Characteristics of IFNγ-sEVs and native sEVs have been shown in our recent study^[24]^. To clarify the effect of IFNγ-sEVs on ONC, we administered the native sEVs or IFNγ-sEVs with a concentration of 10^9^ through intravitreal injection in mice immediately after ONC [Figure 1A]. The results of FVEP showed that the treatment with native sEVs and IFNγ-sEVs significantly improved the FVEP detection ripple compared to the ONC group at 7 days post sEVs injection [Figure 1B]. With further analysis, the prominent decrease in the latency of the N1 wave [Figure 1C] and the significant increase in the amplitude of the P1 wave [Figure 1D] were found in the ONC mice treated by IFNγ-sEVs compared with those of the native sEVs group. Then, we performed immunofluorescence staining of Brn3a and Rbpms to quantify the number of RGCs in the degenerated retina and found that IFNγ-sEVs significantly increased the number of RGCs than native sEVs (Figure 1E, F, G, and H). The trend of H&E staining of retinas was consistent with that of immunofluorescence [Supplementary Figure 2A and B]. Furthermore, the therapeutic effects of IFNγ-sEVs and native sEVs lasted at least 14 days [Supplementary Figure 1C-G]. Overall, the findings indicated that IFNγ-sEVs had enhanced therapeutic effects on ONC-triggered RGC injury compared to native sEVs.

### Pretreatment with IFNγ significantly enhanced the inhibitory effect of MSC-derived sEVs on inflammatory signaling pathways in the ONC mice

**Figure 2:**
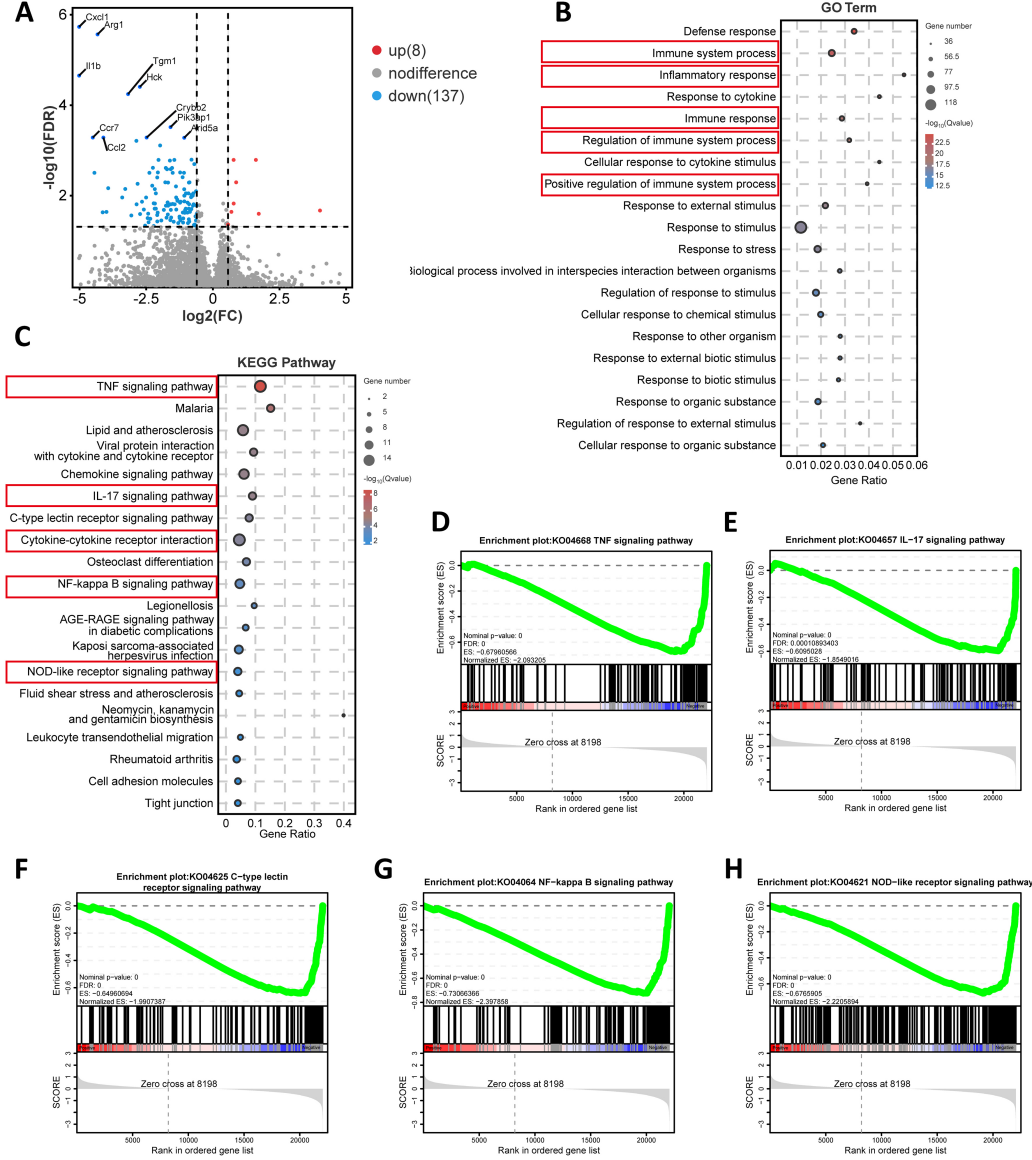
IFNγ-sEVs inhibit the inflammation signal pathway in the retina after ONC. (A) Volcano plot of genes that are highly and lowly expressed in the retina of the IFNγ-sEVs-treated group compared to the native sEVs-treated group (*n* = 3 retinal samples from ONC mice, with adjusted *P*-values ≤ 0.05); (B) GO analysis of DEGs in the IFNγ-sEVs group compared with the native sEVs group; (C) KEGG analysis of DEGs in the IFNγ-sEVs group compared with the native sEVs group; (D-H) Significantly downregulated gene sets associated with inflammation signal pathway in the IFNγ-sEVs-treated group compared with those native sEVs-treated group determined by GSEA. ONC: Optic nerve crush; sEVs: small extracellular vesicles; DEGs: differentially expressed genes; KEGG: Kyoto Encyclopedia of Genes and Genomes; GSEA: Gene Set Enrichment Analysis; GO: Gene Ontology; IFNγ: interferon-gamma.

To elucidate the mechanisms of sEVs alleviating the damage of RGCs, mRNA sequencing was applied to identify DEGs in the untreated retinas after ONC 7 days, as well as in the ONC retinas treated with native sEVs and IFNγ-sEVs [Supplementary Figure 3A]. The results indicated that in the IFNγ-sEVs group, eight DEGs were notably upregulated, while 137 DEGs were significantly downregulated in comparison to the native sEVs group [Figure 2A]. Gene Ontology (GO) enrichment analysis revealed that immune-related items, including immune system processes, inflammatory response, immune response, regulation of immune system processes, and positive regulation of immune system processes, were enriched [Figure 2B]. Furthermore, Kyoto Encyclopedia of Genes and Genomes (KEGG) pathway analysis confirmed significant disparities in inflammatory signaling pathways, such as TNF, IL-17, C-type lectin receptor, NF-kappa B, and NOD-like receptor signaling pathways, significantly differed in the comparison of these two groups [Figure 2C]. Furthermore, Gene Set Enrichment Analysis (GSEA) highlighted that the treatment of IFNγ-sEVs significantly downregulated the above pathways compared with the native sEVs treatment [Figure 2D-H]. In comparison to the ONC group, the native sEVs-treated group exhibited 1,215 DEGs that were upregulated and 110 DEGs that were downregulated, while the IFNγ-sEVs-treated group demonstrated 683 DEGs that were upregulated and 73 DEGs that were downregulated [Supplementary Figure 3B]. Immune-related items such as NOD-like, Toll-like, TNF, and C-type lectin receptor signaling pathways were also identified in the KEGG enrichment analysis comparing native sEVs to the ONC group [Supplementary Figure 3C]. Notably, the ribosome pathway was identified in the KEGG enrichment analysis contrasting IFNγ-sEVs with the ONC group [Supplementary Figure 3D]. In conclusion, it indicated that the administration of sEVs derived from MSCs alleviated RGC degeneration mainly through immunoregulation in the ONC retina, and IFNγ pretreatment might enhance the suppressive effect on inflammatory responses in the ONC retina.

### IFNγ-sEVs significantly inhibited the activation of IRM in the retina of ONC

**Figure 3:**
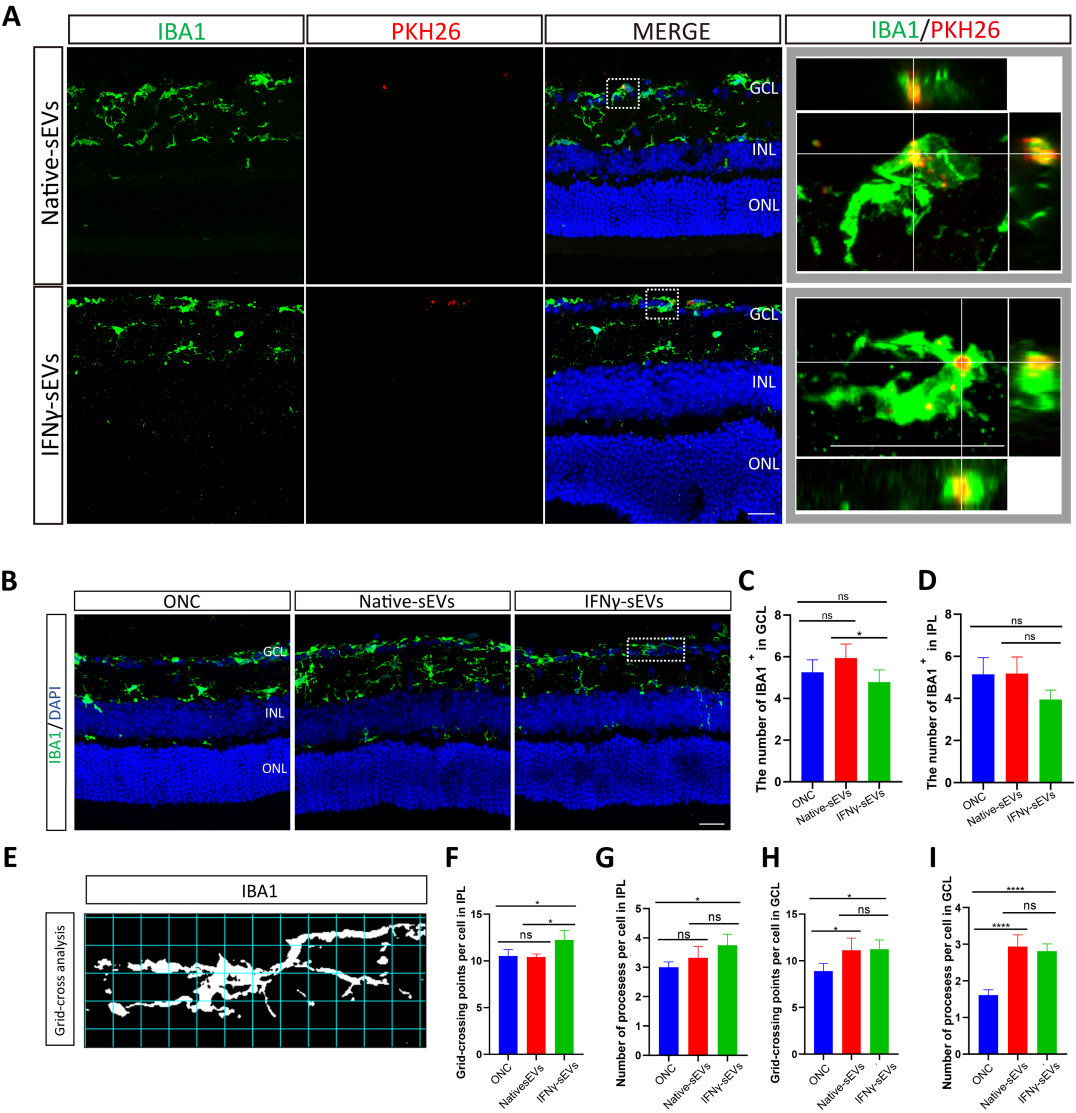
Grafted small extracellular vesicles are internalized by retinal microglia, affecting microglial morphology. (A) The staining of IBA1 (green) in the retinal section of 7 days after injection with PKH26 (red)-labeled sEVs. The rightmost orthogonal diagram of partial enlargement shows sEVs (red) located inside microglia (green). Scale bar 20 μm; (B) Representative immunofluorescence images of IBA1 (green) and DAPI (blue) showing the microglia in the retina across three retinal groups. Scale bar, 20 μm; (C and D) Statistical analysis of the number of IBA1 positive microglia in GCL (C) and IPL (D) counted in confocal images of three groups (*n* = 5 eyes per group); (E) Grid-crossing analysis of representative microglial images; (F-I) Statistical analysis of the average grid-crossing points of microglial cells in IPL (F) or GCL (H), respectively. Statistical analysis of the average processes of microglial cells in IPL (G) and GCL (I), respectively (*n* = 5 eyes per group). Using one-way ANOVA (C, D, F, G, H, and I). Data are shown as mean ± SD. Scale bar: 20 μm. ONC: Optic nerve crush; ONL: outer nuclear layer; IPL: inner plexiform layer; GCL: ganglion cell layer; sEVs: small extracellular vesicles; NS: no significant difference. ^*^P < 0.05; ^**^P < 0.01; ^***^P < 0.001; ^****^P < 0.0001.

Microglia were activated and the number of microglia markedly increased in the retina after ONC in the mice, peaking on day 7^[12]^. PKH26 was utilized to trace the sEVs, while IBA1 served as a specific marker for microglia. It showed that both native sEVs and IFNγ-sEVs were co-localized with IBA1^+^ cells, suggesting sEVs derived from MSCs were able to be internalized by retinal microglia in the GCL [Figure 3A]. No statistical difference in the number of IBA1-positive cells was found between the sEV-treated groups and the control group, although there was a slight reduction in the number of microglia in the GCL following treatment with IFNγ-sEVs compared with those in native sEVs [Figure 3B, C, and D]. Employing standard morphological methods and grid-crossing analysis [Figure 3B and E], it showed that the treatment with two sEVs both led to an increase in grid crossing points and branching sites of microglia in the GCL, while there were no significant differences between IFNγ-sEVs and native sEVs treatment groups [Figure 3F and G]. In IPL, only the IFNγ-sEVs treatment showed an increase in branches and grid crossing points [Figure 3H and I]. In summary, the results suggested that sEVs derived from MSCs contributed to the regulation of microglial activation in the retina of ONC.

**Figure 4:**
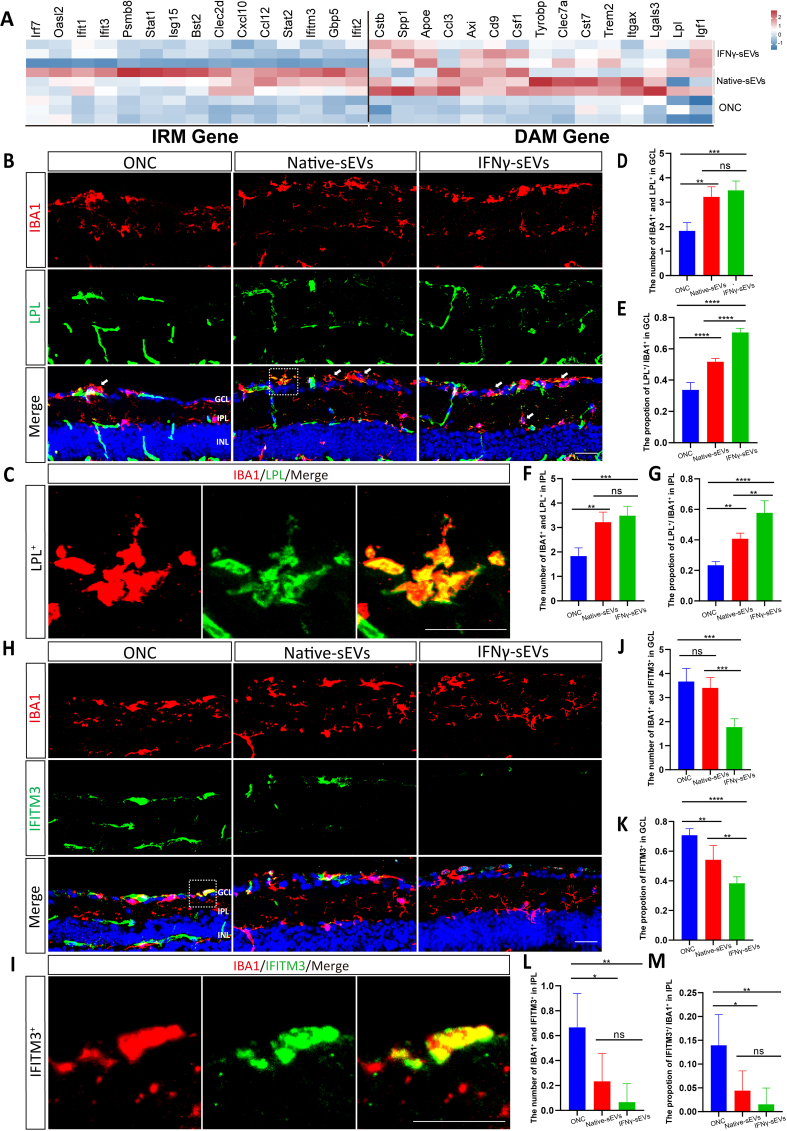
IFNγ-sEVs suppress retinal IFN-responsive microglia activation. (A) Expression heatmap of DAM and IRM typical marker genes in all groups; (B) Immunofluorescent staining of IBA1 (red) and LPL (green) in the retinas of ONC mice treated with sEVs at 7 days were shown in representative high-resolution confocal images. Scale bar, 20 μm; (C) The image exhibits the typical expression of LPL^+^ in the IBA1^+^ cells co-located with sEVs in the retinas at a higher magnification. Scale bar, 30 μm; (D and F) Quantification of the number of LPL^+^ in the IBA1^+^ microglia in GCL (D) or IPL (F) (*n* = 5 eyes per group); (E and G) Quantification of the proportion of LPL^+^ microglia in GCL (E) or IPL (G) (*n* = 5 eyes per group); (H) Immunofluorescent staining of IBA1 (red) and IFITM3 (green) in the retinas of ONC mice treated with sEVs at 7 days are shown in representative high-resolution confocal images. Scale bar, 20 μm; (I) The image shows the typical expression of LPL^+^ in the IBA1^+^ cells co-located with sEVs in the retinas at a higher magnification. Scale bar, 30 μm; (J and L) Quantification of the number of IFITM3^+^ in the Iba1^+^ microglia in GCL (J) or IPL (L) (*n* = 5 eyes per group); (K and M) Quantification of the proportion of IFITM3^+^ microglia in GCL (K) or IPL (M) (*n* = 5 eyes per group). Using one-way ANOVA (D, E, F, G, J, K, L, and M). Data are shown as mean ± SD. ONC: Optic nerve crush; ONL: outer nuclear layer; IPL: inner plexiform layer; LPL: lipoprotein lipase; GCL: ganglion cell layer; IFNγ: interferon-gamma; sEVs: small extracellular vesicles; DAM: disease-associated microglia; IRM: interferon-responsive microglia; IFITM3: interferon-induced transmembrane protein 3; NS: no significant difference. ^*^P < 0.05; ^**^P < 0.01; ^***^P < 0.001; ^****^P < 0.0001.

As DAM and IRM recently highlighted the significant roles in the progression of ONC^[19]^, we determined whether sEVs affected microglial phenotypes. Focusing on marker genes in DAM and IRM activation by mRNA sequencing analysis, the heatmap indicated that two kinds of sEVs treatment both led to the upregulation of DAM genes. Notably, only the treatment with IFNγ-sEVs resulted in marked suppression of IRM gene expression compared to the native sEVs treatment group [Figure 4A]. Lipoprotein lipase (LPL) has been identified as a prospective marker for the DAM phenotype and was characterized by high expression levels in DAM^[21,25]^. Double staining for IBA1 and LPL showed that DAM was mainly located in the GCL and IPL of ONC mice [Figure 4B and C]. The numbers of DAM in the GCL and IPL were both increased in IFNγ-sEVs treated and native sEVs-treated groups. Moreover, the proportion of DAM cells in the GCL was higher in the IFNγ-sEVs treated group compared to the native sEVs-treated group [Figure 4D-G]. Subsequently, interferon-induced transmembrane protein 3 (IFITM3) was selected as a marker of IRM phenotype^[20,26]^, and it showed that the IFITM3-positive microglia were mainly localized in the GCL of ONC mice [Figure 4H and I]. It revealed no statistical difference in IRM numbers between the native sEVs group and the ONC group in GCL. However, a considerable reduction in the number of IRM was observed in the group receiving IFNγ-sEVs [Figure 4J and K]. IRM was infrequently observed in the IPL of the three groups; nevertheless, both sEVs-treated groups exhibited a decrease in the number and proportion of IRM in the IPL compared to the ONC group [Figure 4L and M]. These results indicated that MSC-sEVs promoted DAM activation when administered to the retina of ONC mice. Furthermore, sEVs from MSCs pretreated with IFNγ specifically inhibited the transition of microglia into the IRM phenotype.

### IFNγ-sEVs contributed to microglia activation by regulating inflammatory response and ribosome activity *in vitro*

**Figure 5:**
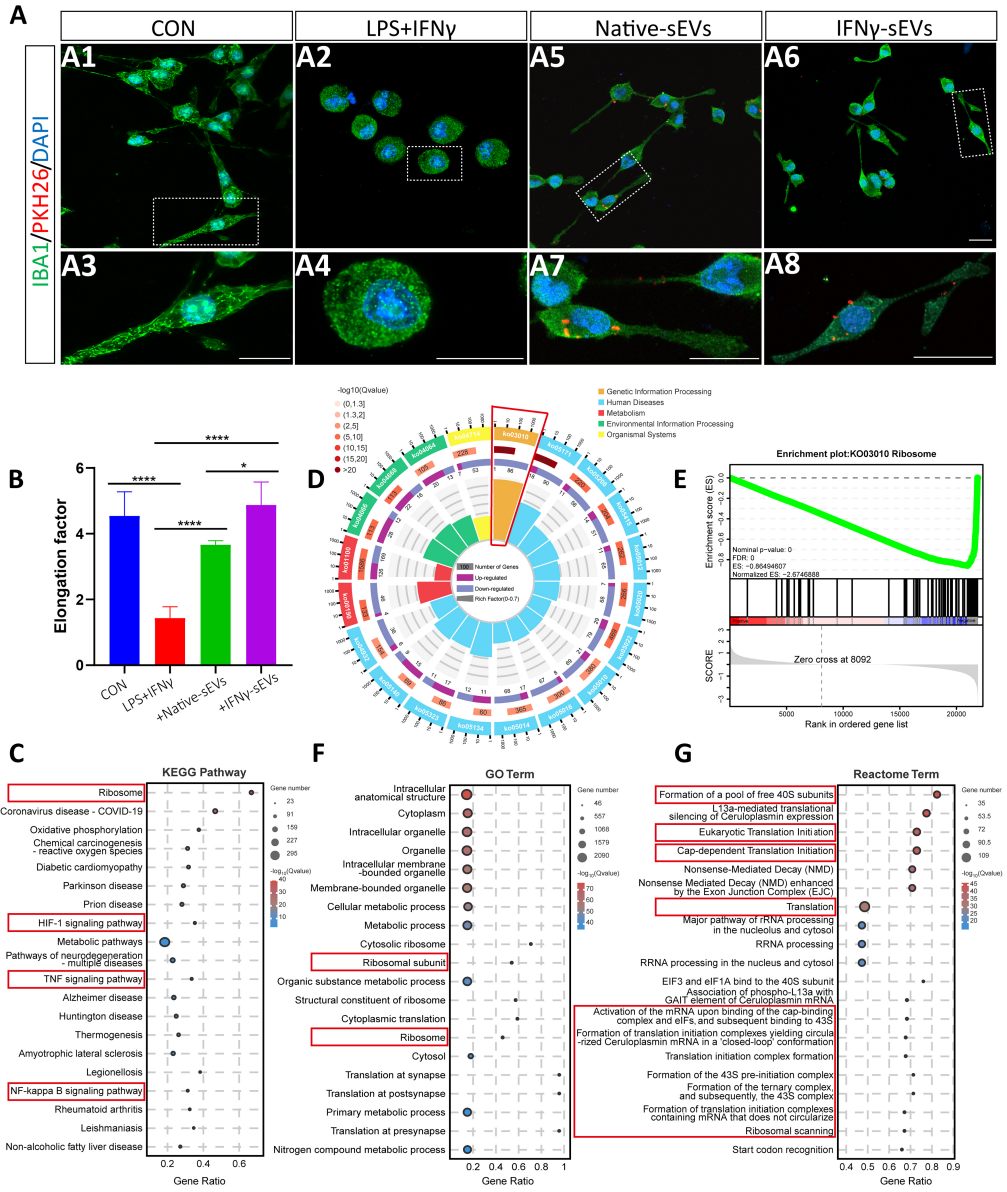
IFNγ-pretreated small extracellular vesicles promote inhibition of microglia activation by regulating microglial inflammatory response and ribosome activity *in vitro*. (A) Representative confocal images depict the morphology of IBA1-stained BV2 cells (green) cultured as negative control (A1), stimulated with LPS + IFNγ for 24 h (A2), and internalized of sEVs (red) labeled with PKH26 24 after LPS + IFNγ-stimulation (A5, A6). The partial enlargement (A3, A4, A7, A8). Scale bar, 20 μm; (B) The quantization of elongation, that is, the length of the long axis divided by the length of the short axis (*n* = 3 samples per group); (C and D) KEGG analysis of DEGs in the IFNγ-sEVs group compared with the native sEVs group; (E) The DEGs of the ribosome pathway in the IFNγ-sEVs group were determined by GSEA; (F) GO analysis of DEGs in the IFNγ-sEVs group compared with the native sEVs group; (G) Reactome analysis of DEGs in the IFNγ-sEVs group compared with the native sEVs group. Using one-way ANOVA (B). IFNγ: Interferon-gamma; LPS: lipopolysaccharide; KEGG: Kyoto Encyclopedia of Genes and Genomes; DEGs: differentially expressed genes; sEVs: small extracellular vesicles; GO: Gene Ontology; NS: no significant difference. ^*^P < 0.05; ^***^P < 0.0001.

To clarify the underlying mechanism by which sEVs from MSCs influenced microglia activation, the BV2 microglia cell line was applied. Microglia stimulated with lipopolysaccharide (LPS) and IFNγ for 24 h showed a significant activation compared to the control group, characterized by an unbranched amoeba-like morphology [Figure 5A1, 2, 3, and 4]. Both native sEVs or IFNγ-sEVs, labeled with a red fluorescent dye (PKH26), demonstrated internalization by LPS + IFN-γ-activated BV2 cells following co-culture for 24 h, resulting in a morphological transition from the activated microglia to a branching state [Figure 5A5, 6, 7, 8]. The native sEVs and IFNγ-sEVs treatments both significantly enhanced the elongation rate of microglia, with IFNγ-sEVs exhibiting a better effect [Figure 5B]. To explore the signaling pathways involved in the modulation of microglial activation by sEVs, RNA sequencing was performed in three groups: stimulated BV2, native sEVs-treated BV2, and IFNγ-sEVs-treated BV2 groups. Primary component analysis revealed significant differences in gene expression profiles among the three groups [Supplementary Figure 4A]. Compared to the stimulated group, 719 DEGs were identified as upregulated and 781 DEGs downregulated in the native sEVs group, while in the IFNγ-sEVs group, 462 DEGs were upregulated and 559 downregulated. Additionally, in the comparison between the IFNγ-sEVs and native sEVs treatments, 2,241 DEGs were identified, with 1,049 upregulated and 1,192 downregulated [Supplementary Figure 4B]. A pairwise comparison among the three groups revealed 224 co-occurring DEGs [Supplementary Figure 4C], and KEGG pathway analysis showed that the DEGs of the three groups were enriched into several inflammation-related pathways including IL-17, TNF, NK-kappa B, and HIF-1 pathways and ribosome pathway [Figure 5C and Supplementary Figure 4D and E], consistent with the results *in vivo* [Figure 2C and Supplementary Figure 3C and D].

**Figure 6:**
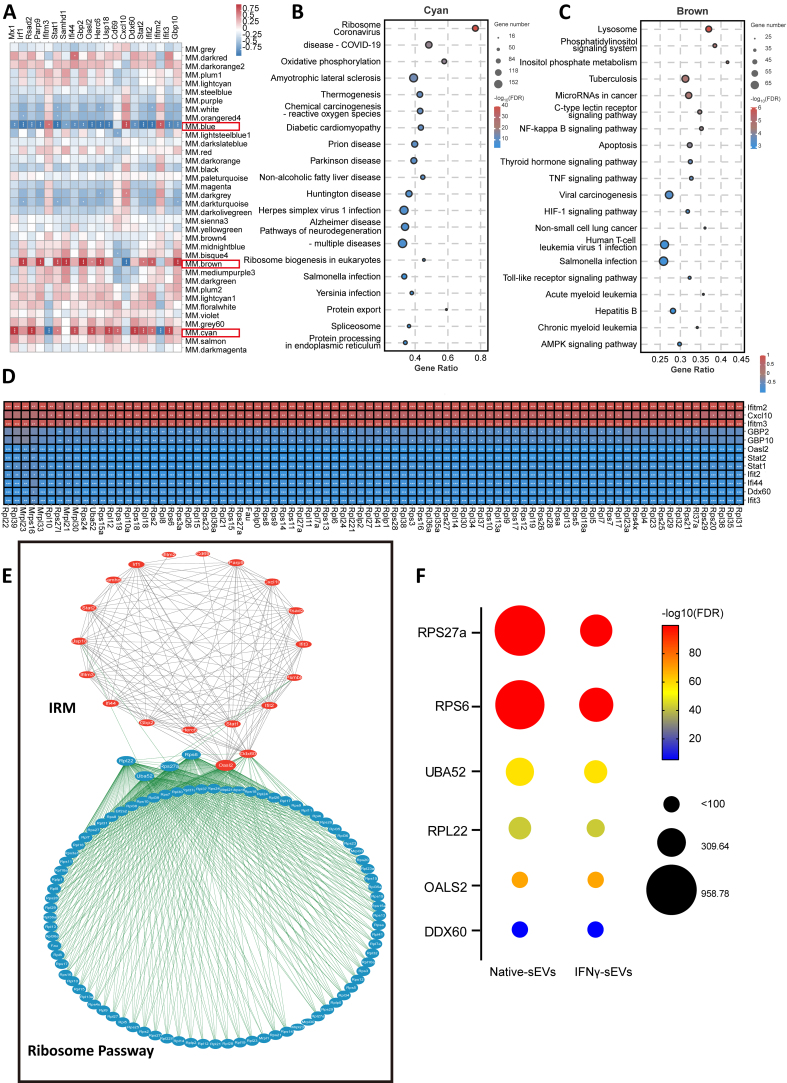
IRM are regulated by the ribosome pathway. (A) Correlation heatmap analyzed the association between modules and differentially expressed IRM genes; (B and C) KEGG Analysis of cyan (B) and brown (C) modules; (D) Correlation heatmap analyzed the association between differentially expressed genes on ribosome pathways and IRM genes; (E) Molecular network of differentially expressed genes on ribosome pathway (blue) and IRM genes (red); (F) The bubble chart shows the expression level and different significance of the genes lying in the center of the molecular network diagram in the native sEVs and the IFNγ-sEVs. IRM: Interferon-responsive microglia; KEGG: Kyoto Encyclopedia of Genes and Genomes; IFNγ: interferon-gamma; sEVs: small extracellular vesicles.

**Figure 7:**
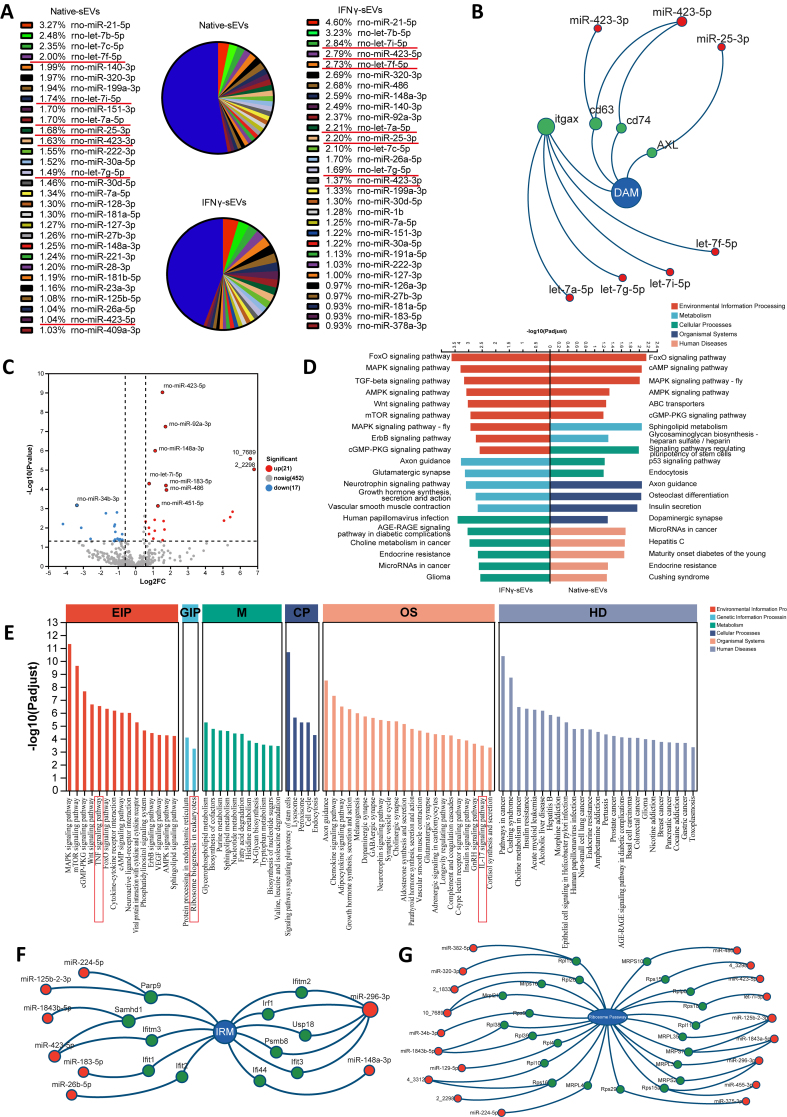
MiRNAs in IFNγ-sEVs regulate gene expression of IRM and ribosome pathways, both directly and indirectly. (A) Pie charts displaying the miRNA abundance in native sEVs and IFNγ-sEVs, as determined by microRNA sequencing. The matching proportions are presented alongside the top 30 most abundant miRNAs (*n* = 3 samples per group), color-coded for clarity; (B) The common mirnas of the top 30 most abundant mirnas in each of the two sEVs, many of which target DAM marker genes; (C) A volcano plot showing statistically significant differentially expressed miRNAs in IFNγ-sEVs compared with native sEVs (adjusted *P*-values ≤ 0.05); (D) KEGG analysis of highly expressed miRNAs in native sEVs and IFNγ-sEVs; (E) KEGG analysis of significantly differentially expressed miRNAs in IFNγ-sEVs compared with native sEVs; (F) The significantly differentially expressed miRNA target maker genes of IRM; (G) The significantly differentially expressed miRNA target ribosomal genes. IFNγ: Interferon-gamma; sEVs: small extracellular vesicles; IRM: interferon-responsive microglia; DAM: disease-associated microglia; KEGG: Kyoto Encyclopedia of Genes and Genomes.

**Figure 8:**
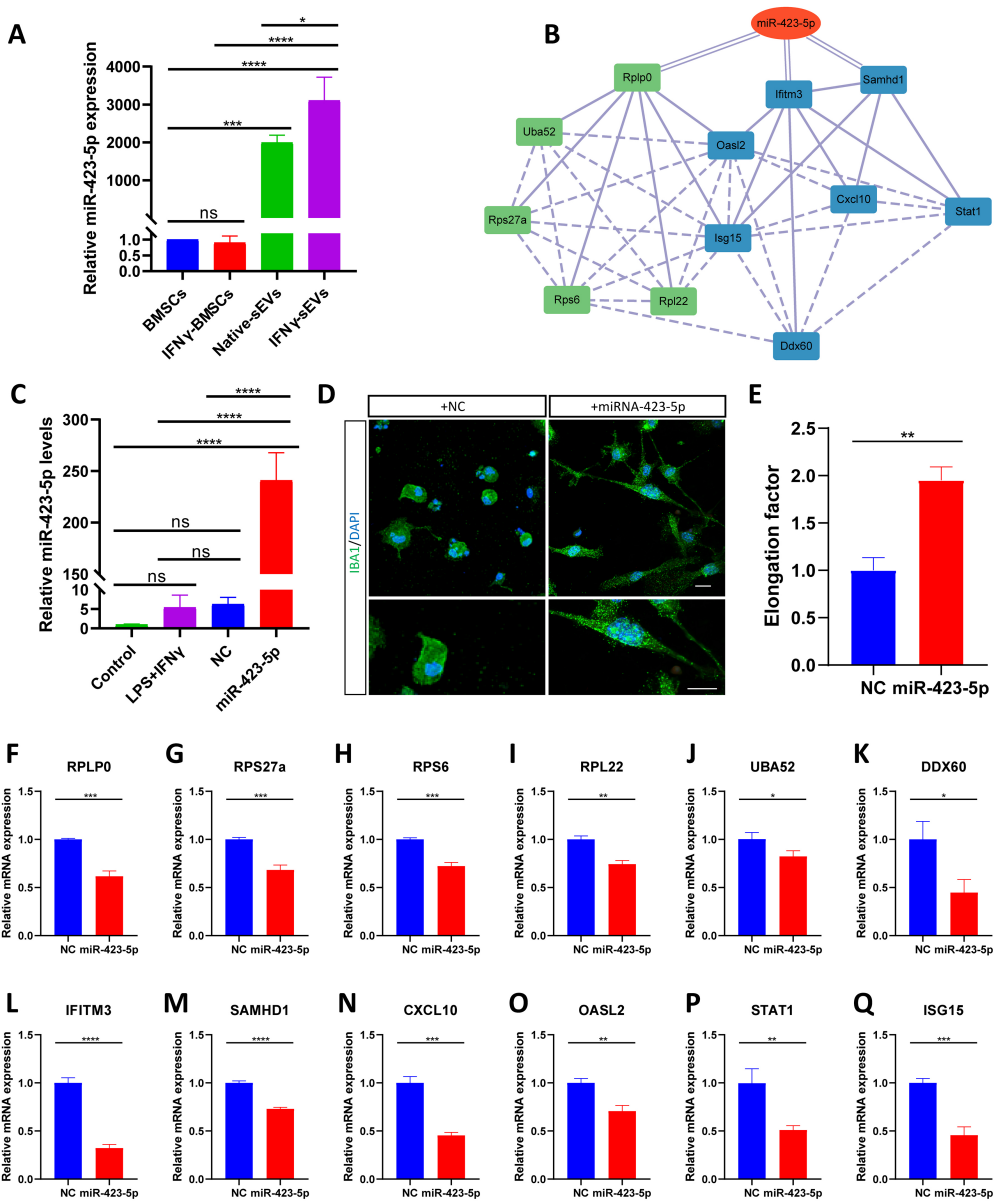
*miR423-5p* suppresses the expression of ribosomal genes and IRM genes. (A) RT-qPCR analysis showing the expression levels of *miR-423-5p* in BMSCs, IFNγ-BMSCs, native sEVs, and IFNγ-sEVs (*n* = 3 samples per group); (B) Molecular network showed *miR-423-5p* related to ribosome-IRM core network and *miR-423-5p* predicted to target *RPLP0*, *IFITM3*, and *SAMHD1*; (C) RT-qPCR analysis showing the levels of *miR-423-5p* in control BV2 cells, BV2 cells stimulated with LPS + IFNγ, and BV2 cells added either NC or *miR-423-5p* after LPS + IFNγ-stimulation (*n* = 3 samples per group); (D) Representative confocal images show the morphology of *IBA1*-stained BV2 cells treated with *miR-423-5p* mimics or NC for 48 h after being stimulated with LPS + IFNγ for 24 h. Scale bar, 20 μm; (E) The quantization of elongation, that is, the length of the long axis divided by the length of the short axis (*n* = 3 samples per group); (F-K) RT-qPCR analysis showing relative mRNA levels of ribosomal genes in the NC group and *miR-423-5p*-treated group (*n* = 3 samples per group); (L-Q) RT-qPCR analysis showing relative mRNA levels of IFN-responsive microglial genes in the NC group and *miR-423-5p*-treated group (*n* = 3 samples per group). Using one-way ANOVA (A and C). Using one-way ANOVA (A and C) and unpaired t-tests (E-Q). Data are shown as mean ± SD. IRM: Interferon-responsive microglia; RT-qPCR: reverse-transcription quantitative polymerase chain reaction; BMSCs: bone marrow mesenchymal stem cells; IFNγ: interferon-gamma; sEVs: small extracellular vesicles; NC: negative control; LPS: lipopolysaccharide; NS: no significant difference. ^*^P < 0.05; ^**^P < 0.01; ^***^P < 0.001; ^****^P < 0.0001.

Notably, changes in the expression of numerous mRNAs encoding ribosomal proteins, including small ribosomal subunit (Rps) and large ribosomal subunit (Rpl), were observed in microglia following sEVs treatment. Specifically, the effect was more pronounced in the IFNγ-sEVs compared to the group of native sEVs. Furthermore, the ribosome pathway was identified as the most significant pathway in the KEGG pathway enrichment analysis of DEGs between the native sEVs and IFNγ-sEVs groups [Figure 5C]. In detail, 67% of genes within the ribosome pathway exhibited significant differences, with 86 DEGs significantly downregulated and 1 DEG significantly upregulated in the IFNγ-sEVs group in contrast to the native sEVs group [Figure 5D]. GSEA analysis further supported the downregulation of the ribosome pathway in the IFNγ-sEVs treatment group [Figure 5E]. The ribosome pathway has also been identified in the GO enrichment analysis comparing IFNγ-sEVs and native sEVs [Figure 5F]. Additionally, Reactome analysis confirmed significant differences between the two treatment groups in the ribosome-related items, including the formation of a poor free 40S subunits pathway, eukaryotic translation initiation pathway, and so on [Figure 5G].

To explore the potential mechanism of special modulation of IRM activation by IFNγ-sEVs, Weighted Gene Co-expression Network Analysis (WGCNA) was used to classify genes exhibiting similar expression patterns. Thousands of genes were classified into 36 modules, revealing a significant association of IRM genes with cyan, blue, and brown modules [Figure 6A]. According to KEGG analysis, the cyan, brown, and blue modules were mainly associated with the ribosome pathway [Figure 6B], the inflammatory response signaling pathway [Figure 6C], and the cellular processes, respectively [Supplementary Figure 4F]. The correlation heatmap analysis further confirmed the strong association between differentially expressed ribosomal genes and those related to IRM genes [Figure 6D]. Rps27a, Rps6, Uba52, Rpl22, Oasl2, and Ddx60 were identified as key genes linking the ribosome pathway and IRM genes [Figure 6E], with most of them displaying downregulated expression in IFNγ-sEVs-treated group [Figure 6F]. This finding suggested that IFN-γ pretreatment enhances the ability of sEVs to regulate ribosome function, thereby facilitating the specific regulation of IRM activation by IFNγ-sEVs.

### IFNγ-sEVs modulated ribosome activity and suppressed IRM activation via miRNAs

MiRNAs have been identified as crucial biological cargoes within MSCs-derived sEVs promoting neuroprotection in injured or degenerated retina^[27]^. We performed miRNA sequencing on native sEVs and IFNγ-sEVs to elucidate the mechanisms by which IFNγ stimulation improved the protective effects of sEVs on injured RGCs [Supplementary Figure 5A]. Correlation analysis revealed that the Pearson correlation coefficient between native sEVs and IFNγ-sEVs ranged from 0.819 to 0.928, indicating a high degree of similarity in miRNA content between the two sEVs types [Supplementary Figure 5B]. Notably, the expression levels of the top 30 miRNAs constituted approximately 50% of the total miRNA expression in both native sEVs and IFNγ-sEVs [Figure 7A]. Among these, 7 common miRNAs were identified that target DAM marker genes [Figure 7B], which may regulate the activation of DAM in the retina of ONC mice [Figure 4B]. A comparative analysis of miRNA composition between IFNγ-sEVs and native sEVs demonstrated that 21 miRNAs were upregulated, while 17 miRNAs were downregulated in IFNγ-sEVs [Figure 7C and Supplementary Figure 5C]. To identify the unique features of miRNAs in the two sEVs types, KEGG enrichment analyses were used to analyze the highly expressed miRNA. It indicated that the pathways related to environmental information processing, metabolism, and cellular processes were enriched in IFNγ-sEVs, whereas organismal systems and human diseases were predominantly enriched in native sEVs [Figure 7D]. Furthermore, KEGG analysis revealed that target genes of these significantly differentially expressed miRNAs were associated with the TNF signaling pathway, IL-17 signaling pathway, and MAPK signaling pathway [Figure 7E], which were classical signals in inflammatory responses and immune regulation. Notably, the ribosome biogenesis in the eukaryotic pathway was also identified [Figure 7E]. Of the 38 differentially expressed miRNAs, eight miRNAs were found to directly target the characteristic genes of IRM [Figure 7F], while 19 miRNAs targeted genes in the ribosomal pathway [Figure 7G]. It suggested a potential mechanism by which IFNγ-sEVs could indirectly and directly suppress IRM activation. In summary, these findings suggested that IFNγ-sEVs were enriched with both inflammation-inhibitive miRNAs and ribosome-inhibitive miRNAs, which may effectively suppress IRM activation in the retina of ONC mice.

Among the 38 differentially expressed miRNAs, *miR-423-5p* exhibited the most statistically significant differences [Figure 7C] and ranked among the top 5 in expression levels within IFNγ-sEVs [Figure 7A]. qPCR analysis was performed to evaluate the expression levels of *miR-423-5p* in BMSCs, IFNγ-BMSCs, native sEVs, and IFNγ-sEVs. The results revealed that *miR-423-5p* levels were higher in IFNγ-sEVs compared to native sEVs, but there was no difference in BMSCs and IFNγ-BMSCs [Figure 8A]. *miR-423-5p* was predicted to target ribosomal genes such as *RPLP0*, as well as IRM genes including *IFITM3* and *SAMHD1*, potentially linking key genes in the ribosome-IRM network [Figure 6E] to modulate its activity [Figure 8B]. We added *miR-423-5p* mimics or negative control (NC) into BV2 cells following stimulation with LPS + IFNγ. The results of qPCR analysis demonstrated that the levels of *miR-423-5p* were significantly increased in BV2 cells treated with the *miR-423-5p* mimics. Conversely, no significant differences in *miR-423-5p* levels were noted among the stimulated BV2 cells, the NC-treated BV2 cells, and the untreated control BV2 cells [Figure 8C]. Immunofluorescence analysis demonstrated significant changes in the morphology of microglia treated with *miR-423-5p* mimics, displaying a ramified pattern [Figure 8D] and enhanced elongation compared to the NC group [Figure 8E]. Additionally, results of reverse-transcription quantitative polymerase chain reaction (RT-qPCR) revealed a marked downregulation in the expression levels of core ribosomal genes (including *RPLP0*, *RPS27a*, *RPS6*, *RPL22*, and *UBA52*) and IRM genes (such as *DDX60*, *IFITM3*, *SAMHD1*, *CXCl10*, *OASL2*, *STAT1*, and *ISG15*) in the *miR-423-5p* mimics-treated group relative to the NC-treated group [Figure 8F-Q]. These findings indicated that IFNγ pretreatment modulated the miRNA expression of sEVs, thereby contributing to the suppression of IRM activation and ribosome activity.

## DISCUSSION

It has been considered that sEVs may have potential therapeutic benefits^[28]^. MSC-derived sEVs demonstrate significant potential as therapeutic agents for the treatment of acute CNS injuries, due to their immunomodulatory and regenerative properties^[15,29]^. In our recent study, we observed an effective strategy to improve the neuroprotective effect of MSC-sEVs on RGC degeneration and clarified the underlying mechanisms in the ONC model. Our results demonstrated that preconditioning MSCs with IFN-γ substantially enhanced the therapeutic efficacy of their sEVs in reducing RGC loss by promoting the anti-inflammatory responses in the retina of ONC. Both native sEVs and IFNγ-sEVs were found to promote DAM activation, while IFNγ-sEVs exhibited a unique capacity to suppress IRM activation during RGC degeneration. Furthermore, mRNA-seq analysis revealed that IFNγ-sEVs exhibited a more pronounced ability to regulate inflammation response and ribosomal function in contrast to native sEVs in BV2 cells. Additionally, the analysis of sEVs’ miRNA sequencing identified specific miRNAs within MSC-sEVs that might play critical roles in modulating the activation of DAM and IRM.

The characteristics of sEVs exhibit variability depending on the condition of the MSCs. Thus, the preconditioning of MSCs with cytokines, hypoxia, and chemicals has been demonstrated to enhance the immunomodulatory and regenerative effects of sEVs effectively^[7]^. Our recent study shows that IFN-γ priming induces MSCs to increase sEVs production with larger diameters and upregulated expression of CD63, CD81, and Cd9^[24]^, consistent with the previous study^[30]^. In the current study, we confirmed that IFN-γ preconditioning enhanced the protective effects of sEVs derived from rat BMSCs on degenerative RGCs at both 7 and 14 days in ONC mice. RNA sequencing analysis of the retina revealed that IFNγ-sEVs exhibited a greater anti-inflammatory effect than native sEVs, with the downregulation of several signaling pathways associated with the TNF, IL-17, NF-kappa B, NOD-like receptor, and C-type lectin receptors. Previous studies have indicated that IFNγ-sEVs markedly reduce the levels of pro-inflammatory cytokines in the LPS-induced models involving human peripheral blood mononuclear cells^[9]^ and splenocytes^[31]^. It suggests that IFNγ-sEVs contain an elevated concentration of anti-inflammatory components, thereby suppressing inflammation. The results of this study implied that the preconditioning of MSCs through the addition of cytokines provided straightforward approaches to manipulating sEVs cargo, which can enhance the therapeutic potential of MSC-derived sEVs in various pathological conditions.

The acute response to CNS inflammation is a multifaceted process that involves an array of immune cells. Among these, microglia are the immunoreactive cells closely associated with the onset and progression of neuroinflammatory responses^[32]^. Notably, the depletion of microglia using PLX5622 demonstrated a transient neuroprotective effect against RGC loss 5 days post ONC^[33]^. However, this depletion failed to enhance the survival of RGCs at a later stage (days 7-21) following ONC^[34]^, suggesting that the microglial activation and subsequent inflammation-mediated neurotoxicity play critical roles in exacerbating RGC death mainly during the early stage of the injury (7 days or less), which corresponds to the peak period of RGC loss. Consequently, our investigation sought to explore the effects of sEVs on microglia activation at 7 days after ONC. The results indicated that a significant majority of sEVs were internalized by microglia in the retina of ONC. Furthermore, it was observed that rounded microglia reverted to a ramified morphology 7 days following the administration of sEVs *in vivo* (24-h incubation of sEVs *in vitro*), whereas the number of microglia in the GCL decreased slightly only with the treatment of IFNγ-sEVs. Microglia with a rounder cell body, typically characterized by fewer and shorter processes, are commonly categorized as “activated” which corresponds with an inflammatory response and the release of inflammatory cytokine interleukin 1β (IL-1β)^[35]^. These morphological changes in microglia suggest underlying functional remodeling. Recent findings have established that MSC-sEVs effectively reduce levels of inflammatory and pro-inflammatory factors in acute CNS injury mice^[15]^, thereby indicating that sEVs may attenuate neuroinflammation in the retina of the ONC model by modulating microglia activation.

sEVs derived from MSC have been found to promote the transformation of “M1” microglia, which are characterized as pro-inflammatory and neurotoxic, into “M2” microglia, known for their anti-inflammatory and neuroprotective characteristics^[15,36]^. However, recent advancements in single-cell technologies have provided compelling evidence that microglia do not polarize into these two types^[17]^. Furthermore, recent studies indicate that microglia can respond to multiple insults and activate diverse phenotypes, which may either exacerbate or ameliorate the pathology of neurodegenerative diseases^[18]^. Consequently, redirecting microglial activation toward beneficial and neuroprotective phenotypes rather than completely removing resident microglia from the injured retina and optic nerve, exhibits the potential to possibly inhibit the progression of RGC degeneration after ONC. In our investigation, we observed that IFNγ-sEVs and native sEVs both increased the number of DAM. Notably, IFNγ-sEVs specifically reduced the number of IRM 7 days after ONC. These findings suggested that MSC-derived sEVs might mediate the phenotypic transformation of microglia, thereby ameliorating acute neuroinflammatory responses and producing neuroprotective effects. DAM activation enhances microglial phagocytosis and lipid metabolism ^[13,37,38]^, ultimately improving RGC survival by eliminating dead cells and cellular debris^[12]^. Conversely, IRM has been identified as a pivotal component in driving the IFN program and acts as the main producer of type I IFN, which leads to synapse loss and neuronal death at the late stage of neurodegeneration^[20,26,39,40]^. Type-I IFN has emerged as a central mediator of neuroinflammation and is implicated in a cascade of toxic consequences in CNS injury^[41,42]^. IRM may emerge in response to cGAS/STING signaling during neurodegeneration^[43]^. Inhibition of microglial cGAS-STING signaling alleviates the loss of RGSs in the retina of ONC^[44]^, suggesting that IRM activation may aggravate the loss of damaged RGCs. Despite these multiple toxic effects of type-I IFN in the CNS, it is also associated with protective immunosuppression and anti-inflammatory effects in the retina^[45,46]^, contributing to nerve regeneration in the ONC model^[10]^. Accumulating evidence indicates that IFNs are highly protective when expressed at the appropriate levels; however, over-production can lead to significant damage. This observation is consistent with the “double-edged sword” hypothesis concerning innate immune activation in neurodegeneration. Therefore, we proposed that interventions, aimed at manipulating the type-I IFN pathway in neurodegeneration, should concentrate on suppressing the excessive production of IFN.

Changes in multiple inflammation-related genes are observed during the development of microglia subtypes^[47]^, providing strong support for the notion that inflammation-related pathways are essential for the induction of disease-stage-specific microglia subtypes following CNS injury^[48,49]^. This study confirmed that specific inflammation-related pathways, including TNF and NF-kappa B, played important roles in the regulation of microglial subtypes by MSC-sEVs *in vitro*, which is consistent with findings from previous studies^[36]^. The RNA-sequencing analysis identified the most significant changes in the ribosome pathway induced by MSC-sEVs treatment in LPS + IFNγ-activated BV2 cells. Ribosomes, as energy-consumptive organelles, are responsible for the translation of mRNA into proteins in a highly regulated process^[50]^. A cluster of highly upregulated injury-induced mRNAs is not translated; instead, selective translational repression leads to the development of distinct microglia molecular signatures^[51]^. A novel ribosome-based checkpoint mechanism has been confirmed to regulate the translation of innate immune genes and microglial activation in the context of neuroinflammation^[51,52]^, indicating that the translation dynamics of microglia ribosomes play a vital role in the post-transcriptional regulation of immune response in resident microglia. In both acute and chronic neurodegeneration, specific microglia subtypes increased the expression of mRNAs encoding ribosomal proteins. Additionally, microglia with a transitory cluster enriched in ribosomal genes have been termed ribosomal microglia (RM), indicating an active turnover of microglia^[53,54]^. Based on these findings, we hypothesized that MSC-sEVs might enhance the microglial capacity to produce phenotype-related proteins and mRNA translation by regulating ribosomal biogenesis, both of which are essential during phenotypic transitions. Significantly, IFNγ-sEVs demonstrated a unique ability to suppress the ribosome pathway when treating the BV2 line. Furthermore, it was confirmed that differentially expressed ribosomal genes were correlated with IRM genes, suggesting that IFNγ-sEVs might inhibit IRM activation by suppressing ribosome activity and protein translation in the ONC model.

IFN-I signaling in microglia is not essential for the transition to DAM, nor does it in turn significantly affect DAM activation^[47]^, indicating that the activation of DAM and IRM occurs dependently. Consequently, we postulated that the different components of sEVs respectively contributed to regulating the phenotypes of DAM and IRM. Recent research indicates that miRNAs are the most critical component of MSC-derived sEVs. These sEVs exert therapeutic effects via miRNA-dependent mechanisms^[55]^, particularly in promoting the neuroprotection of RGCs^[56]^ and modulating neuroinflammation^[57]^. In the present study, we observed that the miRNA contents in IFNγ-sEVs and native sEVs were highly analogous, with both enriched in miR-21-5p, miR-140-3p, miR-151-3p, miR-320-3p, and the let-7 family. These miRNAs are likely to play a significant role in improving the DAM activation. Additionally, IFNγ-sEVs contained 38 miRNAs with significant differential expression, which either directly or indirectly regulated the expression of IRM gens by targeting the genes of the ribosomal pathway. Significantly, *miR-423-5p* was identified as the most significantly upregulated miRNA in IFNγ-sEVs. The upregulation of *miR-423-5p* expression suppresses the polarization of microglia toward the M1 phenotype by downregulating NLR family pyrin domain containing 3 (NLRP3) inflammasomes^[58]^, highlighting its potential as a pivotal regulator in the microglial phenotypic remodeling. *IFITM3*, a marker gene for IRM, is predicted to be a target gene of *miR-423-5p*, playing a vital role in the regulation of IFN-I induction in response to viral stimulation^[59]^. The knockdown of *IFITM3* has been found to suppress the M1-like polarization of microglia^[60]^, suggesting that *miR-423-5p* may directly inhibit IRM activation. Another target gene, *rplp0*, has been implicated in the response to interferons in immune disorders^[61]^, implying that *miR-423-5p* may also indirectly inhibit IRM activation by modulating the ribosomal pathway. These findings indicated that miRNAs in sEVs played a predominant role in the regulation of the microglial phenotypes.

Despite the significant potential of sEVs derived from MSCs for the treatment of ONC, the present study exhibited limitations. Primarily, it is well established that the transcriptomic profiles of microglia *in vitro* cultures are markedly distinct from those in the brain and retina^[62]^. Several DAM and IRM genes, which exhibit high expression levels in the microglia of CNS, display low expression levels in cultured microglia^[62-64]^, explaining our different results of sEVs regulating these genes between *in vivo* and *in vitro*. Unfortunately, a microglial culture system that accurately replicates the state of microglia in the CNS is currently not available^[58]^. Therefore, our findings derived from the BV2 cell line require further validation through microglia obtained from human induced pluripotent stem cells (iPSCs), or should also be assessed *in vivo* using single-cell RNA sequencing. Furthermore, additional investigations are needed to elucidate the molecular mechanisms underlying the actions of these miRNAs in modulating microglial phenotypes, with the potential to integrate special miRNAs into MSC-sEVs, thereby selectively regulating specific subsets of microglia.

In conclusion, our study has clarified the effect of MSC-derived sEVs on the phenotypic transformation of microglia and their contribution to RGC degeneration in ONC mice. We have identified the ribosomal pathway as a novel mechanism by which MSC-sEVs mediated microglial activation. Our results provided compelling evidence that preconditioning with IFN-γ enhanced the beneficial effects of MSC-sEVs on neurodegeneration, thereby offering promising approaches for the treatment of injuries in CNS through the selective regulation of microglial phenotypes.

## DECLARATIONS

### Availability of data and materials

The datasets used and analyzed during the current study are available from the corresponding author upon reasonable request. The retinal mRNA sequencing, BV2 cell mRNA sequencing, and miRNA sequencing data are included in the Additional Data 1, 2, 3.

### Copyright

© The Author(s) 2025.
