# Supplementary material for: IFNγ preconditioning improves neuroprotection of MSC-derived vesicles on injured retinal ganglion cells by suppressing microglia activation via miRNA-dependent ribosome activity
Source: Extracell Vesicles Circ Nucl Acids. 2025 Feb 19;6(1):87–111. doi: 10.20517/evcna.2024.66 (PMC11977360; doi:10.20517/evcna.2024.66)
Supplement: Supplementary file 1 [file evcna-6-1-87-SupplementaryMaterials.pdf]

Supplementary Materials

IFNγ preconditioning improves neuroprotection of MSC-derived vesicles on injured retinal ganglion cells by suppressing microglia activation via miRNA-dependent ribosome activity

Tianjing You[1,2,3,#], Yuanxing Yang[2,3,#], Luodan A[2,3,#], Xuan Cheng[2,3], Xi Lin[2,3], Qingle Liang[2,3], Lingling Ge[2,3], Jing Xie[2,3], Siyu Chen[2,3], Na Liu[2,3,] Juncai He[2,3,4], Haiwei Xu[2,3], Xiang Ma[1]

[1]Department of Ophthalmology, The First Affiliated Hospital of Dalian Medical University, Dalian 116014, Liaoning, China.
[2]Southwest Eye Hospital, Southwest Hospital, Third Military Medical University (Amy Medical University), Chongqing 400038, China.
[3]Key Lab of Visual Damage and Regeneration and Restoration of Chongqing, Southwest Eye Hospital, Southwest Hospital, Chongqing 400038, China.
[4]Department of Ophthalmology, The 920 Hospital of PLA Joint Logistics Support Force, Kunming 650032, Yunnan, China.
[#]Authors contributed equally.

Correspondence to: Prof. Xiang Ma, Department of Ophthalmology, The First Affiliated Hospital of Dalian Medical University, No. 9 West Section of Lushun South Road, Dalian 116014, Liaoning, China. E-mail: Xma9467@vip.sina.com; Prof. Haiwei Xu, Southwest Eye Hospital, Southwest Hospital, Third Military Medical University (Amy Medical University), No. 29 Gaotanyan Main Street, Shapingba District, Chongqing 400038, China. E-mail: xuhaiwei@tmmu.edu.cn; Dr. Juncai He, Department of Ophthalmology, The 920 Hospital of PLA Joint Logistics Support Force, No. 212, Daguan Road, Xishan District, Kunming 650032, Yunnan, China. E-mail: hejuncai@tmmu.edu.cn

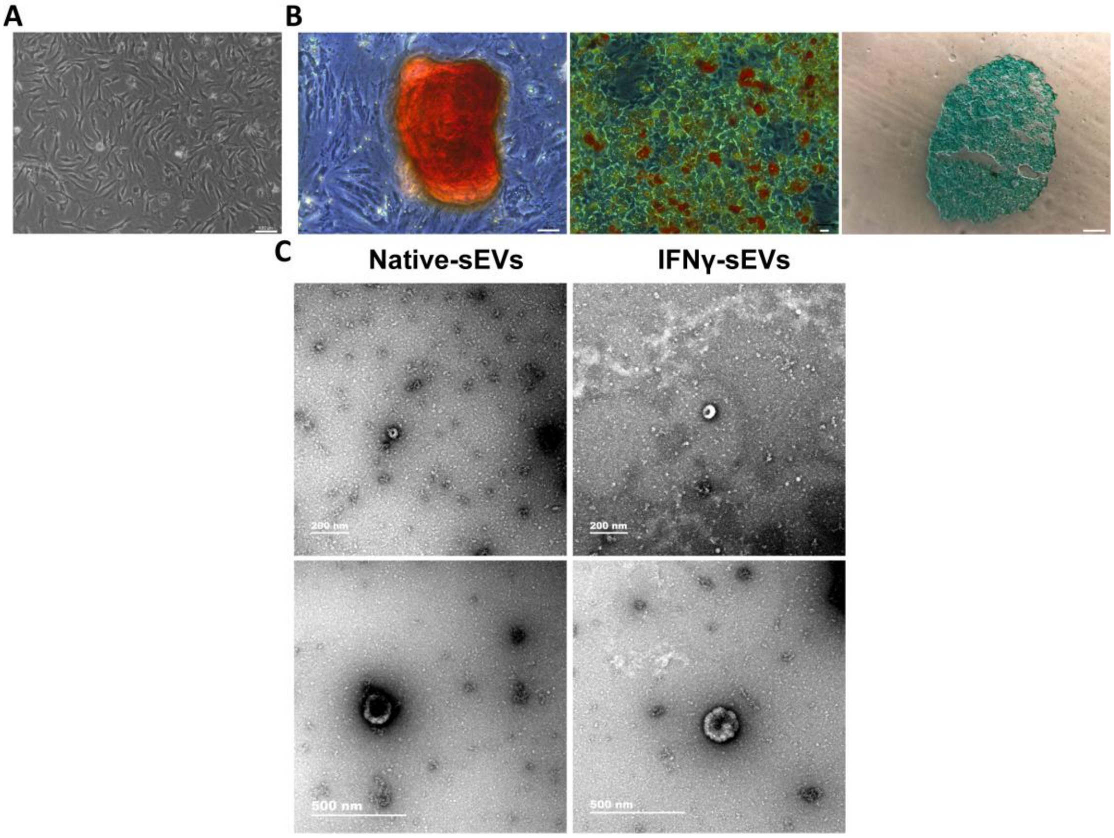

Supplementary Figure 1. Identification of bone marrow mesenchymal cells and small extracellular vesicles. (A) Morphologic map of bone marrow mesenchymal cells in the third generation. Scale bar, 100 μm; (B) Representative images of bone marrow mesenchymal cell differentiation potential: osteogenic differentiation; lipogenic differentiation; chondrogenic differentiation (from left to right). Scale bar, 100 μm. C. Typical morphology of Native-sEVs and IFNγ-sEVs under TEM (n = 3 samples per group).

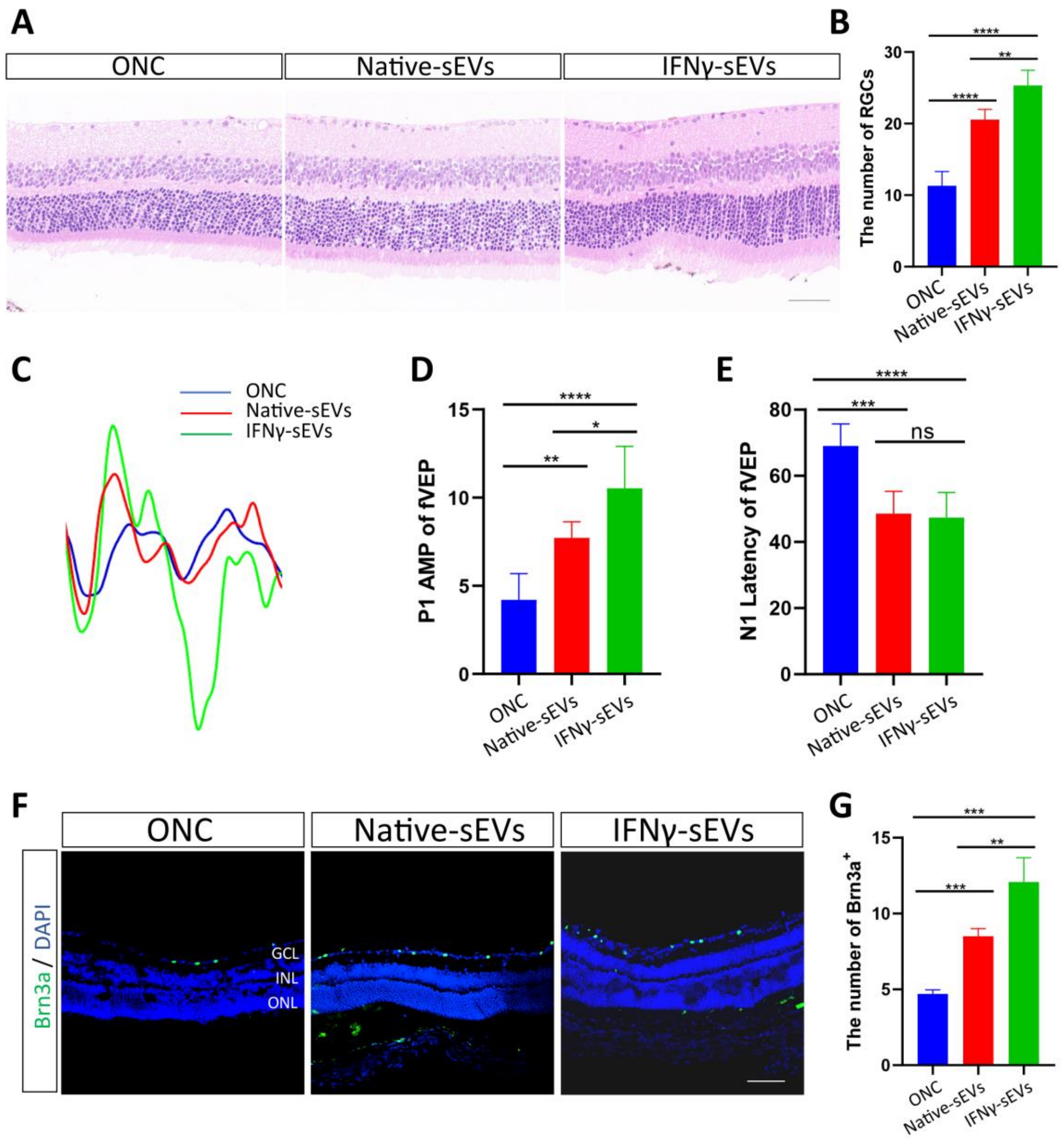

Supplementary Figure 2. Implantation of small extracellular vesicles derived from IFNγ-primed MSCs preserves visual function and RGC counts after ONC 14 days. (A) Representative H&E staining images of retinal sections 7 days after sEVs transplantation. Scale bar, 50 μm; (B) Graph showing the mean number of RGCs per field (*n* = 5 eyes per group); (C) Representative waveforms of FVEP test 14 days after sEVs implantation; (D and E) P1 amplitudes (D) and N1 latencies (E) at 14 days after ONC and sEVs treatment (*n* = 5 eyes per group); (F) Representative images of retina from sEVs infusion after ONC, immunohistochemically stained for Brn3a (green) and DAPI (blue). Scale

bar, 20 μm; (G) Graph depicting the mean number of Brn3a positive RGC in a region of retina either side of the optic nerve head (n = 3 eyes per group). ONC: Optic nerve crush; FVEP: flash visual evoked potential; ONL: outer nuclear layer; IPL: inner plexiform layer; GCL: ganglion cell layer. NS: no significant difference, $^{*}P < 0.05$, $^{**}P < 0.01$, $^{***}P < 0.001$, $^{****}P < 0.0001$. Using one-way ANOVA (B, D, E and G). Data are shown as mean ± SD.

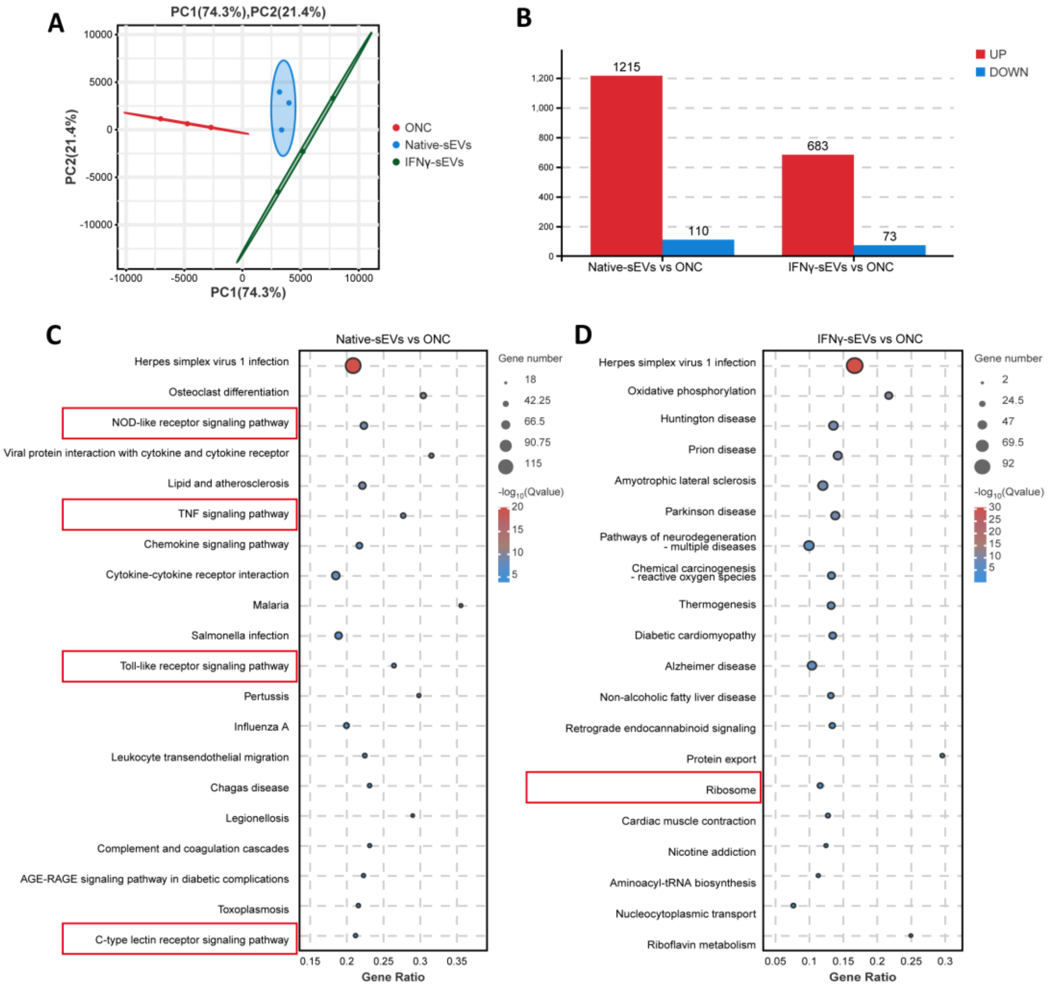

Supplementary Figure 3. RNA sequencing results of the Native-sEVs treated retina group and the IFNγ-sEVs treated retina group compared with the ONC group respectively. (A) Primary component analysis; (B) The bar graph shows the number of up-regulated and down-regulated DEGs in the Native-sEVs treated retina group and the IFNγ-sEVs treated

retina group compared with ONC group respectively; (C) KEGG analysis of DEGs in the Native-sEVs treated retina group compared with ONC group; (D) KEGG analysis of DEGs in the IFNγ-sEVs treated retina group compared with ONC group.

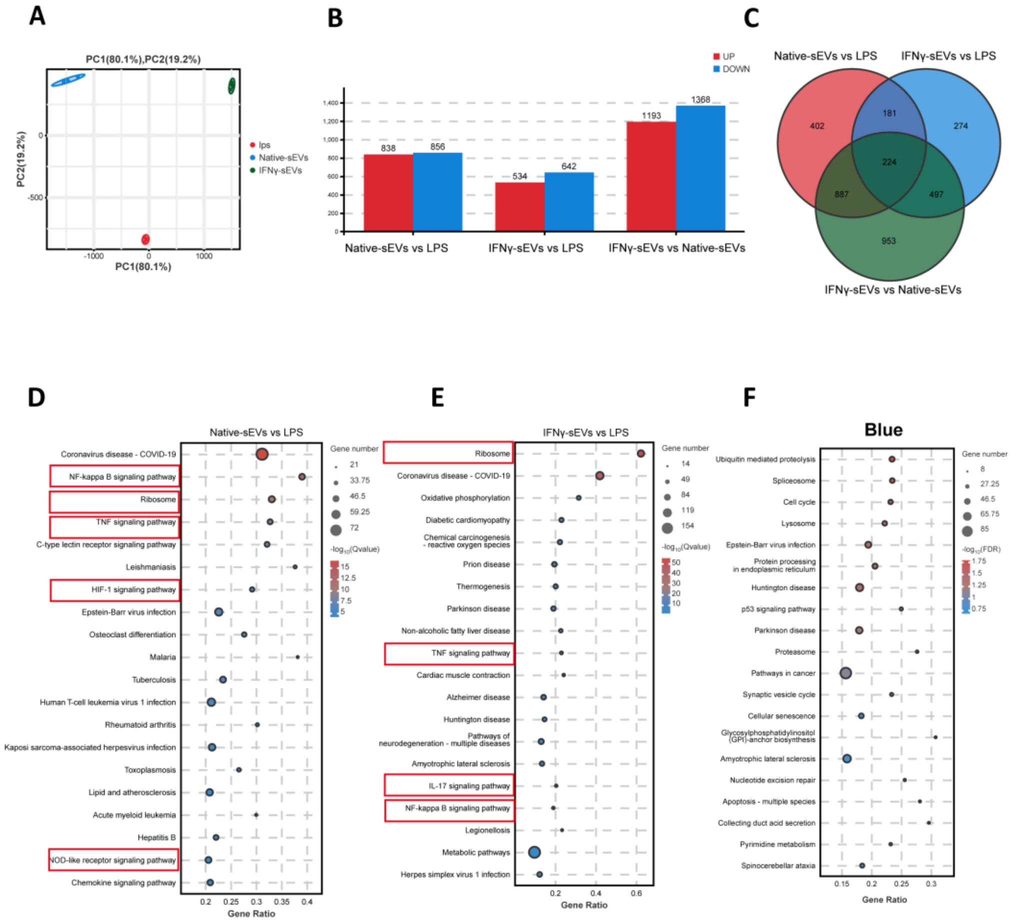

Supplementary Figure 4. RNA sequencing results of Native-sEVs treated BV2 group and IFNγ-sEVs treated retina group compared with stimulated BV2 group respectively. (A) Primary component analysis; (B) The bar graph shows the number of up-regulated and down-regulated DEGs in the pairwise comparison of the three groups; (C) Venn diagram of three groups of differential genes; (D) KEGG analysis of DEGs in the Native-sEVs treated BV2 group compared with stimulated BV2 group; (E) KEGG analysis of DEGs in the IFNγ-sEVs treated BV2 group compared with stimulated BV2 group; (F) KEGG analysis of the blue module.

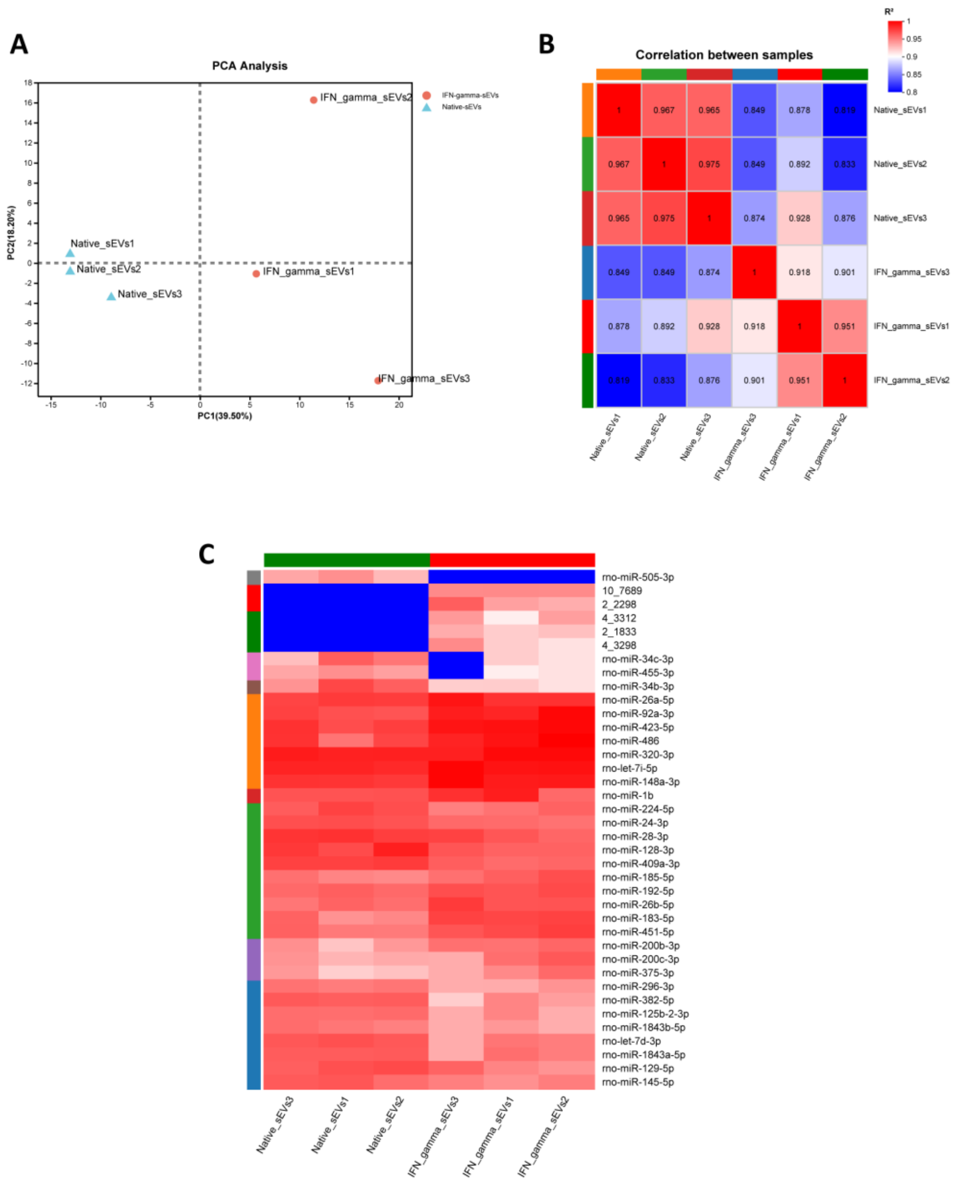

Supplementary Figure 5. Comparison of miRNA sequencing results between Native-sEVs and IFNγ-sEVs. (A) Primary component analysis; (B) Heatmap of correlation coefficient from samples; (C) Heatmap of all miRNA differentially expressed in the IFNγ-sEVs compared with the Native-sEVs.

Supplementary Table 1. Antibodies used for immunofluorescence staining

Antibody	Company	Titer	Species	Cat#
Brn3a	Abcam	1:400	Rabbit	ab245230

IBA1	Wako Chemicals	1:500	Rabbit	019-19741
LPL	Abcam	1:200	Mouse	ab21356
IFITM3	R&D Systems	1:200	Goat	AF3377

Supplementary Table 2. Primers used for RT-qPCR

**Name**	**Primer-F**	**Primer-R**
miR-423-5p	TGAGGGGCAGAGAGCGAG	AGTGCAGGGTCCGAGGTA
RPLP0	TGGGCAAGAACACCATGATG	CGGATATGAGGCAGCAGTTTC
RPS27a	AGGCCAAGATCCAGGATAAGG	CACCACCACGAAGTCTCAACA
RPS6	CGAGGAGCCATAGTGAGGAAC	GGAGCATAAAAATCCACCAGGA
RPL22	CATGGCAGTGAGAGACCATAATG	AGTGGCAATAGACCTTCTCCATA
UBA52	GACCCTTACGGGGAAAACCAT	AGACAAAGTCCGGCCATCTTC
DDX60	ACTGGAACACTCGCTTTGG	GAAGTAGACATCACCCAACAGG
IFITM3	CCCCCAAACTACGAAAGAATCA	ACCATCTTCCGATCCCTAGAC
SAMHD1	GAGAATCGTGGTTTCCGAGAG	CTCCAAGGAACTTACTCCCAGA
CXCL10	TCTGAGTGGGACTCAAGGGA	TCAACATGCGGACAGGATAG
OASL2	CAAGAAGTCAGGGTGATTAAGG	GAACAGAATCATGTCTTGGTCA
STAT1	GGCGAAGAGCGACCAGAAAC	AGCTGATCCAGGCAGGCATT
ISG15	CCACCAAAGCGGTGAAATGC	GACTTGCTCGTGCTGGGACA
GAPDH	AAGGTCGGTGTGAACGGATT	TGAACTTGCCGTGGGTAGAG
